# Stretchable, dynamic covalent polymers for soft, long-lived bioresorbable electronic stimulators designed to facilitate neuromuscular regeneration

**DOI:** 10.1038/s41467-020-19660-6

**Published:** 2020-11-25

**Authors:** Yeon Sik Choi, Yuan-Yu Hsueh, Jahyun Koo, Quansan Yang, Raudel Avila, Buwei Hu, Zhaoqian Xie, Geumbee Lee, Zheng Ning, Claire Liu, Yameng Xu, Young Joong Lee, Weikang Zhao, Jun Fang, Yujun Deng, Seung Min Lee, Abraham Vázquez-Guardado, Iwona Stepien, Ying Yan, Joseph W. Song, Chad Haney, Yong Suk Oh, Wentai Liu, Hong-Joon Yun, Anthony Banks, Matthew R. MacEwan, Guillermo A. Ameer, Wilson Z. Ray, Yonggang Huang, Tao Xie, Colin K. Franz, Song Li, John A. Rogers

**Affiliations:** 1 Center for Bio-Integrated Electronics, Northwestern University, Evanston, IL 60208 USA; 2 Querrey Simpson Institute for Biotechnology, Northwestern University, Evanston, IL 60208 USA; 3 Department of Materials Science and Engineering, Northwestern University, Evanston, IL 60208 USA; 4 Department of Bioengineering, University of California, Los Angeles, Los Angeles, CA 90095 USA; 5 Division of Plastic and Reconstructive Surgery, Department of Surgery, National Cheng Kung University Hospital, College of Medicine, National Cheng Kung University, Tainan, 70456 Taiwan; 6 International Research Center for Wound Repair and Regeneration, National Cheng Kung University, Tainan, 70456 Taiwan; 7 School of Biomedical Engineering, Korea University, Seoul, 02841 Republic of Korea; 8 Interdisciplinary Program in Precision Public Health, Korea University, Seoul, 02841 Republic of Korea; 9 Department of Mechanical Engineering, Northwestern University, Evanston, IL 60208 USA; 10 Department of Medicine, University of California, Los Angeles, Los Angeles, CA 90095 USA; 11 State Key Laboratory of Structural Analysis for Industrial Equipment, Dalian, University of Technology, 116024 Dalian, China; 12 Department of Engineering Mechanics, Dalian University of Technology, 116024 Dalian, China; 13 International Research Center for Computational Mechanics, Dalian University of Technology, 116024 Dalian, China; 14 State Key Laboratory of Chemical Engineering, College of Chemical and Biological Engineering, Zhejiang University, 310027 Hangzhou, China; 15 Department of Biomedical Engineering, Northwestern University, Evanston, IL 60208 USA; 16 State Key Laboratory of Mechanical System and Vibration, Shanghai Jiao Tong University, 200240 Shanghai, China; 17 Center for Developmental Therapeutics, Chemistry Life Processes Institute, Northwestern University, Evanston, IL 60208 USA; 18 Department of Neurological Surgery, Washington University School of Medicine, St. Louis, MO 63110 USA; 19 Center for Advanced Molecular Imaging, Northwestern University, Evanston, IL 60208 USA; 20 School of Advanced Materials Science and Engineering, Sungkyunkwan University (SKKU), Suwon, 16419 Republic of Korea; 21 Center for Advanced Regenerative Engineering, Northwestern University, Evanston, IL 60208 USA; 22 Department of Surgery, Feinberg School of Medicine, Northwestern University, Chicago, IL 60611 USA; 23 Department of Civil and Environmental Engineering, Northwestern University, Evanston, IL 60208 USA; 24 Regenerative Neurorehabilitation Laboratory, Biologics, Shirley Ryan AbilityLab, Chicago, IL 60611 USA; 25 Department of Physical Medicine and Rehabilitation, Feinberg School of Medicine, Northwestern University, Chicago, IL 60611 USA; 26 The Ken & Ruth Davee Department of Neurology, Feinberg School of Medicine, Northwestern University, Chicago, IL 60611 USA; 27 Department of Neurological Surgery, Feinberg School of Medicine, Northwestern University, Chicago, IL 60611 USA

**Keywords:** Biomedical materials, Implants, Electronic devices

## Abstract

Bioresorbable electronic stimulators are of rapidly growing interest as unusual therapeutic platforms, i.e., bioelectronic medicines, for treating disease states, accelerating wound healing processes and eliminating infections. Here, we present advanced materials that support operation in these systems over clinically relevant timeframes, ultimately bioresorbing harmlessly to benign products without residues, to eliminate the need for surgical extraction. Our findings overcome key challenges of bioresorbable electronic devices by realizing lifetimes that match clinical needs. The devices exploit a bioresorbable dynamic covalent polymer that facilitates tight bonding to itself and other surfaces, as a soft, elastic substrate and encapsulation coating for wireless electronic components. We describe the underlying features and chemical design considerations for this polymer, and the biocompatibility of its constituent materials. In devices with optimized, wireless designs, these polymers enable stable, long-lived operation as distal stimulators in a rat model of peripheral nerve injuries, thereby demonstrating the potential of programmable long-term electrical stimulation for maintaining muscle receptivity and enhancing functional recovery.

## Abstract (web summary)

Bioresorbable electronic stimulators can deliver electrical stimulation in rodents to enhance functional muscle recovery after nerve injury. Here, the authors present a bioresorbable dynamic covalent polymer that enables reliable, long-lived operation of soft, stretchable devices of this type.

## Introduction

Peripheral nerve injuries are among the most devastating causes of sensorimotor deficits, resulting from loss of axonal continuity, death of neuronal cells, and denervation effects on motor, sensory, and autonomic functions^1,2^. Treatments for such injuries involve surgical operations that repair nerve discontinuity with sutures or bridge with nerve grafts or conduits. Even with such interventions, sometimes combined with pharmacological therapies, recoveries can be unpredictable, with unsatisfactory functional outcomes^3^ because (1) the associated muscle groups undergo progressive denervation atrophy, until neuromuscular reinnervation becomes unreceptive to the sprouting axons that regenerate across the gap^4^, and (2) the rates of regeneration of nerves from lesion sites to denervated targets are slow (~1 mm/day)^5^, corresponding to prolonged recovery times and poorer functional prognosis for more proximally located nerve injuries^6^.

Clinical evidence indicates that electrical stimulation of the denervated muscles via transcutaneous electrodes or implantable devices might improve muscle preservation and functional outcome^7,8^. Transcutaneous approaches fail, however, to effectively stimulate deep muscle tissue and they, therefore, are unable to achieve synergic muscle group contraction. Although direct nerve interfaces based on implantable cuff electrodes offer comparatively high levels of stimulation efficiency^9^, discomfort, pain, costs and complications can follow from surgical procedures required to retrieve the devices after a period of use^10,11^. For both transcutaneous and implantable platforms, the supporting hardware typically involves hard wired interfaces and batteries, as inconveniences^9,12–14^ that often lead to non-compliance. As an alternative, our recent work showed that wireless, battery-free, and bioresorbable electronic stimulators can deliver recurring proximal electrical stimulation in rodent models of nerve injury through the initial phases of healing, with enhancements in neuroregeneration and improvements in functional muscle recovery^15^. Non-ideal mechanical properties and relatively short-lived constituent materials, however, limit the lifetimes of these types of devices to timeframes (<6 days) that can be insufficient for nerves to surpass discontinuities associated with many traumatic injuries. Also, the proximal placement of these types of devices provides no direct benefit in reducing muscle atrophy because electrical stimulation at these locations only promotes the onset of motor axon regeneration, without increasing its regeneration speed^16^.

Here, we report (1) materials, device architectures, and integration strategies as the basis of an implantable bioresorbable electrical stimulation platform for traumatic peripheral nerve injuries that features soft, elastomeric mechanics and long-lived materials, and (2) a stimulation strategy, demonstrated in rodent models, that alleviates muscle atrophy resulting from denervation through the application of a bioresorbable cuff electrode interface at a location distal to the nerve injury. A critical biotechnology aspect of these systems is in a specially synthesized bioresorbable dynamic covalent polyurethane (b-DCPU) that serves as a substrate and biofluid barrier for electronics with designs that exploit deformable filamentary serpentine interconnects^17–20^. The elastomeric mechanics of these materials and their low levels of swelling in biofluids facilitate effective use as interfaces with soft tissues/organ systems that exhibit large natural ranges of motion. Electrical stimulation of sciatic nerves in rats repetitively over 30 days demonstrates long-term stability of operation over timeframes relevant to recovery from traumatic nerve injuries. Use of these platforms for multiple episodes of electrical stimulation on the distal nerve stump in a rodent model of peripheral nerve injury (critical nerve gap of 10 mm) indicates an ability to alleviate muscle atrophy arising from denervation. Significant increases in muscle weight, gait function, the size of the regenerated muscle fibers and the number of reinnervated neuromuscular junctions compared to those of a control group suggest that this treatment paradigm, fundamentally enabled by this long-lived bioresorbable system, facilitates neuromuscular recovery, via prevention of early muscle denervation process.

## Results and discussion

### Bioresorbable materials, stretchable designs, and wireless, battery-free operation

**Fig. 1:**
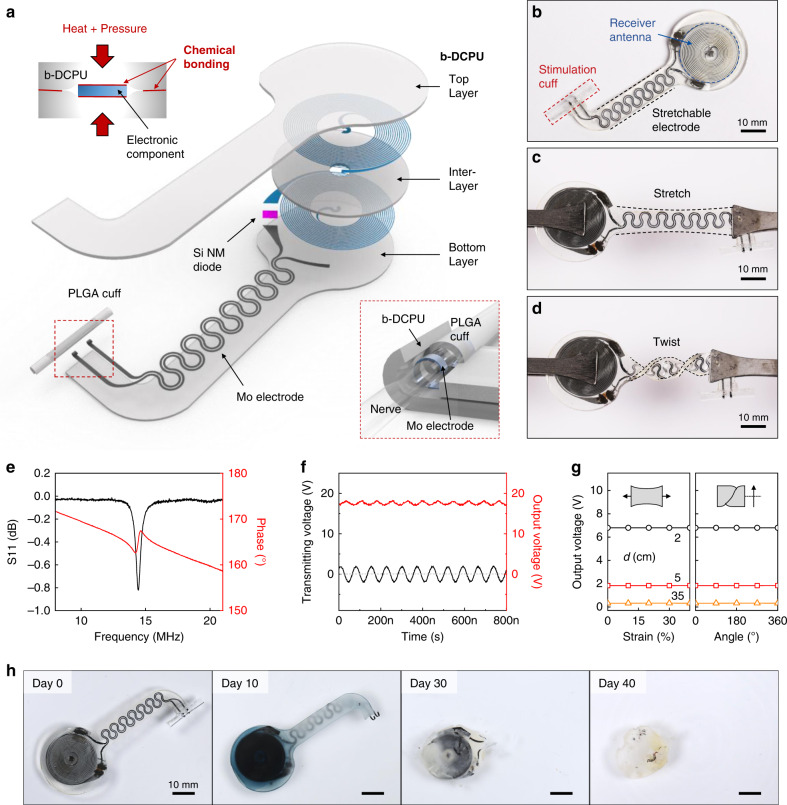
Designs and properties of a long-lived, stretchable, and wireless bioresorbable electrical stimulator to enhance recovery from peripheral nerve injuries. **a** Schematic illustration of the device design. The electronic component consists of three functional parts: (i) a wireless receiver that acts as a radiofrequency power harvester and control interface, built with an inductive coil (Mo, 50 μm thick), a radiofrequency diode (Si NM active layer, 1.2 μm thick), and an interlayer (bioresorbable dynamic covalent polyurethane (b-DCPU), 50 μm thick); (ii) stretchable extension electrodes with serpentine structures (Mo, 15 μm thick with a 200 μm width); and (iii) a stimulation cuff (poly(lactic-co-glycolic acid) (PLGA), 30 μm thick) with exposed electrodes at the ends as an interface to the nerve. All parts of the system, excluding the stimulation cuff, are sandwiched between two layers of bioresorbable elastomers (b-DCPU, 100 μm). The schematic illustrations in the inset show the thermal and stress-activated bonding process for b-DCPU (top, left) and the contact between the nerve and the stimulation cuff (bottom, right). **b** Optical Image of a completed device. **c**, **d** Photographic images of stretched (30%) and twisted (360°) devices. **e** Radiofrequency behavior of the stimulator (black, S11; red, phase). The resonance frequency of ~16.0 MHz allows magnetic coupling in a frequency regime with little parasitic absorption by biological tissues. *n* = 3 independent samples. **f** Example output waveform (stimulator, red) wirelessly generated by an alternating current (sine wave) applied to the transmission coil (transmitter, black). *n* = 3 independent samples. **g** Output voltage of a device as a function of tensile strain (left) and twist angle (right) at different distances between the harvester and transmitter (black, 2 cm; red 5 cm; orange, 35 cm). *n* = 3 independent samples. **h** Images of accelerated dissolution of a bioresorbable wireless stimulator associated with immersion in PBS (pH = 7.4) at 90 °C.

A schematic illustration of a device that highlights the radiofrequency power harvester, stretchable extension electrode and electrical interface to the targeted peripheral nerve appears in Fig. 1a. The bioresorbable dynamic covalent polyurethane (b-DCPU) material offers mechanical stretchability and minimal swelling in biofluids, thereby enabling robust operation in moving tissues, without the limitations on operational lifetimes associated with previously reported bioresorbable nerve stimulators^15^. In particular, without using any external adhesive, the thermal and stress-induced bonding processes generate strong adhesion (i.e., chemical bonding) (1) between the top and bottom layers of the b-DCPU (~100 μm thick) by interfacial bond exchange reactions and (2) between the b-DCPU and other inorganic bioresorbable components by reactions of hydroxyl groups, resulting in minimization of the risk of biofluid penetration at the interface (Fig. 1a, top left inset). The bioresorbable harvester includes a loop antenna in a bilayer, dual-coil configuration (Molybdenum (Mo), ~50 μm thick) with a stretchable b-DCPU as a dielectric interlayer and a radiofrequency diode based on a doped membrane of monocrystalline silicon (1.2 μm thick). A strip of bioresorbable Mo (15 μm thick, 200 μm wide) in a serpentine geometry with an opening at the end serves as an electrical extension and connection to deliver electrical stimuli from the receiver antenna to the nerve tissue. The electrical interface consists of exposed Mo electrodes that encircle the nerve (Fig. 1a, bottom right inset) and a tubular structure of poly(lactic-co-glycolic acid) (PLGA; 75:25 (lactide:glycolide); ~30 μm thick) with a slit along the length of one side to facilitate surgical application over and around the nerve.

Figure 1b shows a photograph of the complete system (width: ~22 mm; length: ~60 mm; thickness: ~200 μm; weight: 370 mg), and Fig. 1c, d highlights the soft, elastic mechanics through pictures of the device during stretching (30%) and twisting (360°), respectively. Figure 1e, f summarizes the electrical performance characteristics. Radiofrequency (RF) power supplied to a transmission antenna placed near the harvester delivers electrical power to the interface with the nerve (Supplementary Fig. S1a). This type of inductive wireless power transfer scheme is common in battery-free medical impants^21,22^, where the use of magnetic coupling (Fig. 1e and Supplementary Fig. S1b, c; ~16.0 MHz) avoids significant RF absorption by biofluids or biological tissues^23^. Figure 1f illustrates monophasic output (red, 17.4 V) for continuous radiofrequency (RF) power (black, ~3.5 V_pp_ at a 1.5 mm coupling distance without load resistor) applied to the transmission antenna (transmitting frequency = 14.8 MHz). Further modulation of the RF power with a low-frequency signal, typically 20 Hz for nerve stimulation, yields cathodic, monophasic electrical impulses to stimulate the target nerve. (Detailed results of electrical performance characterization of the wireless power transfer system are in Supplementary Note 1). The output voltage remains largely unchanged during stretching (Fig. 1g, left) and twisting (right), with little dependence on tensile strain (left) and twisting angle (right). The voltage does, however, depend on the transmitted power (Supplementary Fig. S1e) and the distance between the transmission and receiver coil (Supplementary Fig. S1f). Specifically, increasing the distance between the coils from 1.5 mm to 11 mm decreases the output voltage from 17 V to 2 V for a fixed transmission voltage of 16.6 V_pp_. At distances of 20 mm, output voltages of 2 V, corresponding to typical thresholds for nerve activation, can be generated by a transmitting voltage of 20 V_pp_ at the nerve. For use in large animal models^24^ and in humans^25^ the receiver unit can be located superficially under the skin, with the deformable interconnect structure as an interface to the stimulation site.

**Fig. 2:**
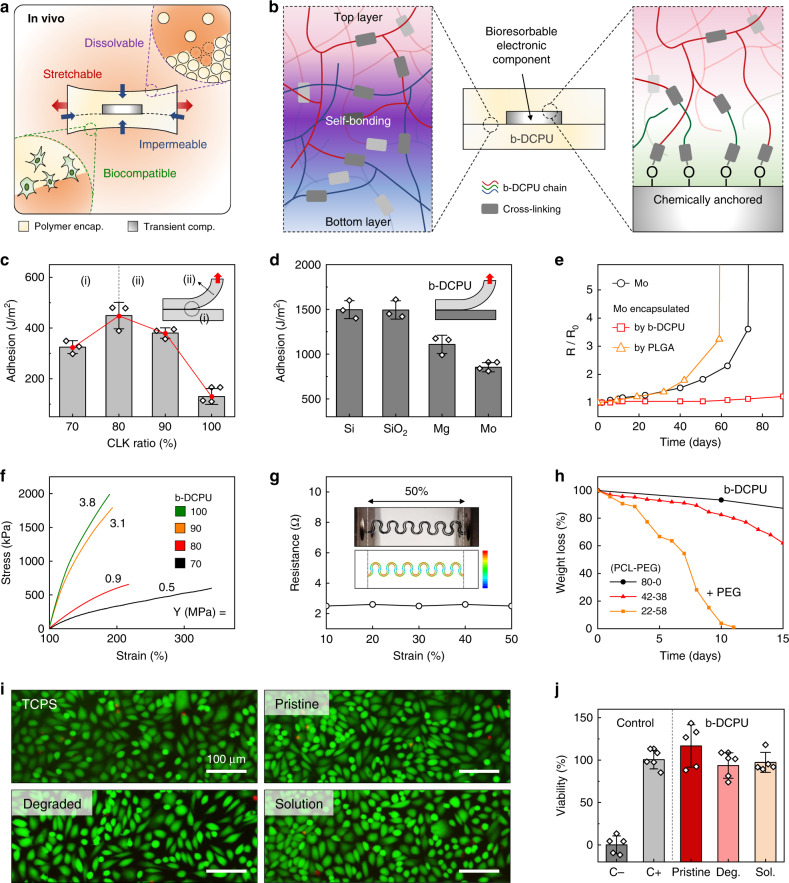
Mechanical, electrical, and biological characterization of a bioresorbable, stretchable substrate, and encapsulation material. **a** Schematic illustration of the key requirements for the materials: dissolvability (i.e., bioresorbability), mechanical stretchability, impermeability against biofluids, and biocompatibility. Adhesion properties. **b** Schematic illustration of the adhesion mechanism associated with the stretchable bioresorbable dynamic covalent polyurethane (b-DCPU) and its self-bonding behavior induced by thermally activated dynamic bond exchange reactions (left); chemically anchored b-DCPU on an inorganic bioresorbable component by covalent bonding (right). **c** Adhesion energy between bonded pieces of b-DCPU with different crosslinking ratios (70, 80, 90, and 100). *n* = 3 independent samples. The inset schematic illustrates contributions from the (i) interfacial energy and (ii) cohesion energy. **d** Adhesion energy between b-DCPU 80 and other inorganic bioresorbable materials, including Si, SiO_2_, Mg and Mo, after the bonding process. *n* = 3 independent samples. Biofluid Impermeability. **e** Changes in resistance of a Mo electrode without encapsulation (black circle), with b-DCPU 80 (red square), and poly(lactic-co-glycolic acid) (PLGA) 65:25 encapsulation (orange triangle) as a function of the immersion time in PBS (pH 7.4) at 37 °C. Mechanical stretchability. **f** Stress–strain curves for b-DCPU materials with different crosslinking ratios (olive, b-DCPU 100; orange, b-DCPU 90; red, b-DCPU 80; black, b-DCPU 70). **g** Changes in resistance of serpentine Mo electrodes encapsulated in b-DCPU 80 during uniaxial stretching (up to the maximum strain of ~50%). The inset shows a photograph and FEA results of b-DCPU-encapsulated Mo serpentine electrodes under uniaxial tensile strains of 50%. The rainbow color scale bar indicates the simulated strain values, from 0% (blue) to 0.6% (red). In vitro dissolution kinetics. **h** Measured changes in weight as a function of the immersion time of b-DCPU 80 (black circle) and PEG containing b-DCPUs (polycaprolactone (PCL): polyethyleneglycol (PEG) = 42:38, red triangle; PCL:PEG = 22:58, orange square) in PBS (pH 7.4) at physiological temperature (37 °C). In vitro biocompatibility**. i** Biocompatibility of various b-DCPU samples in live/dead staining assays of healthy mouse fibroblasts (L929) after 3 days of culture: tissue culture polystyrene (TCPS); normal b-DCPU 80 (Pristine); Degraded b-DCPU 80 (Degraded or Deg.) corresponds to gel-textured b-DCPU; fully dissolved b-DCPU 80 in PBS (Solution or Sol.). *n* = 5 repeated independently with similar results. **j** Normalized in vitro viability assay data. *n* = 5 independent samples. In **c**, **d**, and **j** the results are shown as means ± s.e.m. Data available in source data file.

The essential defining characteristic of this system is that the constituent materials bioresorb in a controlled manner and within a relevant timeframe when exposed to biofluids found in and around subcutaneous tissue. Figure 1h shows photographs of devices collected at various times following immersion in phosphate-buffered saline (PBS, pH 7.4) solution at an elevated temperature (90 °C) for accelerated testing. The materials largely dissolve within 40 days, and all remaining residues completely disappear after 50 days. The rate of degradation of the b-DCPU can be increased by adding hydrophilic polyethyleneglycol (PEG) in appropriate amounts (details are in Fig. 2h).

### Adhesion and mechanical properties of b-DCPU

As shown in Fig. 2a, extending the in vivo operational lifetime of bioresorbable electronic stimulators from a few days to several months demands solutions to daunting challenges in materials science and device design that follow directly from requirements that the system must be (1) electrically reliable in operation, with minimal permeation of biofluids and parasitic leakage of current^26–30^, (2) mechanically robust under various deformations, including stretching, twisting and bending, during natural motions^31^, (3) completely dissolvable (i.e., bioresorbable), at the materials level, without adverse effect^32^, and (4) entirely biocompatible during and after the treatment duration^23^. The operational lifetimes reported here are significantly longer than those of previously reported flexible or stretchable bioresorbable devices, where the operation is limited typically to a few days (Supplementary Note 2)^15,33–35^. Achieving long-lived, stable function relies critically on the b-DCPU and its dynamic covalent networks structure for direct self-bonding^17–20^, as well as surface chemical approaches to ensure robust adhesion to other constituent materials. The synthesis of the b-DCPU involves step-growth polymerization of polycaprolactone-triol (PCL-triol) with hexamethylene diisocyanate (HDI). For both the polymerization and the bond exchange reaction, Tin(II) 2-ethylhexanoate (Sn(Oct)_2_) serves as an FDA-approved biocompatible catalyst^36–38^. Subsequent thermal curing (60 °C) generates a three-dimensional (3D) network structure (Supplementary Fig. S2). (Information on the synthesis procedures and materials properties are in the Methods section and Supplementary Note 3, respectively.) Robust self-bonding between layers of b-DCPU arises mainly from chemical mechanisms via thermally activated dynamic bond exchange reactions (transesterification and transcarbamoylation) (Fig. 2b (left) and Supplementary Figs. S5a and S6)^18,20^. (Information on the adhesion mechanism is in Supplementary Note 4). Two layers of b-DCPU bonded in this manner show interfacial toughnesses approaching 500 J/m^2^ (Fig. 2c). Increasing the crosslinking density reduces the interfacial energy corresponding to the density of chemical bonding by decreasing the number of free hydroxyl groups and the relative concentration of ester moieties, and it increases the cohesion energy. Tailored formulations (isocyanate:hydroxyl ratios of 0.8:1) balance these two factors in an optimal way. Oxygen plasma treatment applied to other inorganic materials allows bonding to b-DCPU via reactions of surface hydroxyl groups in a process that involves hot pressing with uncured precursors (Fig. 2b (right) and Supplementary Fig. S5b). The results of Fig. 2d indicate that strong adhesion (>850 J/m^2^) can be achieved in this manner between b-DCPU and other inorganic bioresorbable materials, including Si, SiO_2_, Mg, and Mo. These robust interfacial bonds lead to long-term electrical stability in simulated physiological conditions. Test structures of Mo electrodes encapsulated in b-DCPU show negligible changes in electrical properties over 80 days in PBS (pH 7.4; 37 °C). By comparison, otherwise similar structures formed with PLGA encapsulation exhibit notable changes over 60 days, primarily limited by weak interfacial adhesion (Supplementary Note 5). The mechanics of b-DCPU are also favorable for the present applications. Depending on the density of crosslinks, the Young’s modulus for this polymer lies between 0.5 and 3.8 MPa (Fig. 2f). Test samples can stretch to more than 170% of their original length, with elastic behaviors for strains larger than 25% (Supplementary Note 6). Serpentine traces of Mo (Supplementary Fig. S8) encapsulated in b-DCPU show unchanged resistances during stretching (ranging from 0–50%) (Fig. 2g) and under cyclic loading (uniaxial stretching to 20% and compression to 20%, involving buckling; 5 mm/s rate; 10,000 cycles) (Fig. S9 and Supplementary Note 6.). These collective results highlight the properties of b-DCPU that allow devices to operate for extended periods in a stable fashion. The rate of bioresorption of the b-DCPU can be controlled over time periods from a week to several months by modifying the composition with the addition of hydrophilic PEG. As shown in Fig. 2h, the b-DCPU degrades slowly, while PEG containing b-DCPU degrades much faster for similar simulated physiological conditions (PBS; pH 7.4; 37 °C) (Supplementary Note 7). The in vitro biocompatibility of b-DCPU samples with three different degradation stages is comparable to that of the control medium (tissue culture polystyrene, TCPS), showing no observable decrease in the in vitro viability of mouse fibroblasts after 3 days culture (Fig. 2i, j and Supplementary Note 8).

### In vivo operation of the bioresorbable electrical stimulators

The low modulus and elastomeric mechanics of this implantable system represent features that significantly decrease the probability for mechanical failure during in vivo operation since tissues, such as skin, muscle, and peripheral nerves, experience strains as large as 20%, corresponding to centimeter-scale displacements during routine postural movement^31^. The mechanical stability of the integrated system can be explored by finite element analysis (FEA), as shown in Supplementary Fig. S14 for deformations associated with stretching, twisting, and out of plane bending. For all of these and other cases of relevance for in vivo operation, the calculated maximum strains in the constituent materials lie below thresholds for plastic deformation or fracture.

**Fig. 3:**
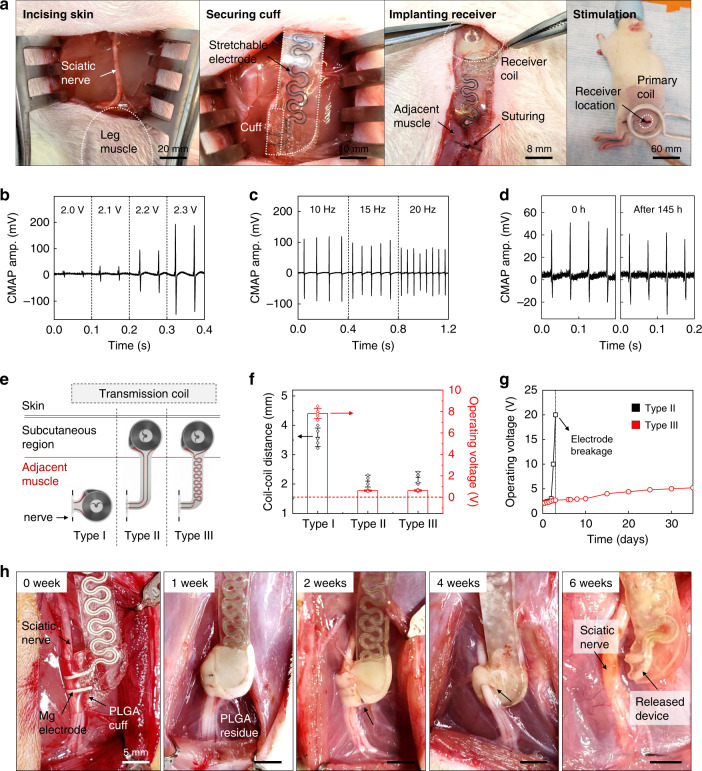
Surgical implantation, operation, and acute demonstration of a long-lived, stretchable, and wireless bioresorbable electrical stimulator for the sciatic nerve in a rodent model. **a** Surgical procedure for implanting the device. From left to right: the skin is incised; the electrical stimulation cuff is introduced on the normal nerve; the radiofrequency harvester unit connected by stretchable extension electrodes is subcutaneously implanted to minimize movement; the skin is sutured and the stimulation is activated with a transmitting coil. **b**, **c** Compound muscle action potential (CMAP) amplitude measured from the gastrocnemius muscle while stimulating the sciatic nerve at various voltages (2.0–2.3 V; at 20 Hz) and frequencies (10, 15, 20 Hz; at 2.2 V). Independent devices (*n* = 10) in independent animals (*n* = 10). **d** Measured changes in CMAP amplitude generated by electrical stimulation at a frequency of 20 Hz after the surgery (0 h) and after 145 h of implantation. Independent devices (*n* = 5) in independent animals (*n* = 5). **e** Schematic illustration of the three different electrodes designs and the position of implantation: Type I device without extension electrode; Type II device with b-DCPU 80 encapsulated straight extension electrodes; and Type III device with b-DCPU 80 encapsulated serpentine extension electrodes. The wireless receiver antennas of Type II and III mount on the subcutaneous region; Type I resides in the muscle adjacent to the nerve. **f** Distance between the harvester and transmitter coils after implanting devices in rats (black dots) and minimum operating voltage required to induce muscle twitching for the different device designs (red bars). Data are presented as means ± s.e.m. *n* = 5 independent samples. **g** Minimum operating voltage required to induce muscle twitching as a function of time (black, Type II; red, Type III). *n* = 3 independent samples. **h** Images of PLGA cuff electrodes on the sciatic nerve for 6 weeks. (*n* = 3 independent animals per groups.) These images illustrate the release of the sciatic nerve from the bioresorbable stimulator after a therapeutic period. Data available in source data file.

Testing of operational stability in animal models involves surgical implantation of the stimulators through a dorsolateral gluteal-muscle-splitting incision to expose the sciatic nerve, as shown in Fig. 3a. Wrapping the cuff around the nerve and securing the interface forms a tubular structure with excellent apposition to the nerve tissue. Inserting the harvester into a subcutaneous pocket created on the dorsolateral aspect of the hindlimb and securing the harvester on top of the gluteal fascia with bioresorbable sutures completes the implantation. Three-dimensional computed tomography images of mice collected 1 week after post-surgery confirm the proper positioning of the receiver and nerve cuff (Supplementary Fig. S15). Passing radiofrequency power through a transmission antenna placed adjacent to the hindlimb delivers electrical stimulation to the nerve, with a temporal pattern defined by modulation of power delivery to the antenna. Electromyograms (EMGs) obtained from the gastrocnemius muscle in uninjured animals confirm the ability to stimulate the sciatic nerve at levels well above threshold. These values, as previously reported^39^, correspond to stimulation of all the nerve fibers via the electrodes placed around the target sciatic nerve. Figure 3b–d shows compound muscle action potential (CMAP) data obtained from stimulation of the sciatic nerve with different voltages (at a frequency of 20 Hz) and different frequencies (with an applied voltage of 2.2 V) using monophasic pulses shortly after the surgery and after 145 h of implantation, respectively.

The stretchable system-level properties enabled by the serpentine Mo structures and the soft b-DCPU encapsulation are critically important to robust, long-lived operation. Experiments with stimulators that adopt three different designs for the Mo electrodes (Fig. 3e and Supplementary Fig. S16)—(1) without extension (Type I), (2) with the linear extension (Type II), and (3) with the serpentine extension (Type III)— demonstrate the essential effects. The minimum operating voltages (i.e., transmitting voltage of primary coil) for muscle twitching, collected from rats with these three different devices (*n* = 3 for each), indicate that the Type I device requires a much higher operating voltage of ~4 V compared to that of the others (~2 V), simply because placing the receiver of the Type I device underneath the skin increases the distance to the transmission coil (Fig. 3f and Supplementary Fig. S16). In all cases, the devices undergo deformations that involve relative displacements/rotations of the receiver and the nerve cuff, due to the natural movements of the rat and the muscle near the sciatic nerve. As shown in Fig. 3g (black), Type II device exhibits electrical failure after 2 days of implantation due to mechanical fracture at the point of load concentration. Failure analysis (Supplementary Fig. S17) suggests that the receiver and nerve cuff parts represent the weakest points for the devices with the Type I and II designs. By contrast, the Type III device shows reliable operation without failure for more than 30 days (Fig. 3g, red; Supplementary Fig. S17), relevant to both animal studies and potential use in humans (e.g., 10 days for rats with 1 cm nerve gap and 30 days for humans with 3 cm nerve gap). Relatively low-operating voltages (~2 V) with negligible changes over time during stimulation (Fig. 3g) are consistent with tight encapsulation without significant leakage.

Designs that decouple the nerve cuff interface from the nerve through processes of degradation after the therapeutic period eliminate continuing mechanical loads on the tissue imparted through the electrical interconnect structure^40^. Supplementary Fig. S18 illustrates how the nerve cuff interface decouples naturally from the nerve as a consequence of bioresorption. The image collected at 20 weeks after implantation shows that the PLGA cuff undergoes bioresorption and that the b-DCPU/Mo/b-DCPU electrode structure remains. (The white appearance is consistent with absorbed water as part of the biodegradation process.) The image at 30 weeks reveals that complete bioresorption of the PLGA decouples the nerve from the b-DCPU encapsulated device. An analogous device designed with a fast rate of bioresorption highlights this decoupling process. Specifically, Fig. 3h shows the behavior of a device constructed with a PLGA 50:50 (lactide:glycolide) cuff and Mg electrode on the sciatic nerve, over a period of 6 weeks. The image at 4 weeks after implantation indicates some residue from the PLGA cuff, with complete bioresorption of the PLGA and Mg at 6 weeks to electrically and mechanically decouple this part of the system from the b-DCPU encapsulated extension electrode. In this way, these designs release the sciatic nerve from the bioresorbable electronic stimulator after a therapeutic period (i.e., functional lifetime) to allow for dissolution of remaining bioresorbable components with relatively slow degradation rates (i.e., degradation time) across extended timescales without adverse mechanical loads on the nerve or immediately surrounding tissues.

Studies of the b-DCPU encapsulation in a rat model demonstrate its biocompatibility. Histological assessments of dorsal subcutaneous implantation show no identifiable immune cells related to b-DCPU encapsulation (Supplementary Fig. S19). Supplementary Fig. S20a shows minimal differences in changes in body weight associated with b-DCPU implanted mice compared with a control group. Analysis of complete blood counts and blood chemistry tests also indicate no sign of organ damage or injury, and no change in the electrolyte or enzyme balance (Supplementary Fig. S20b, c). Detailed results of biocompatibility evaluations are described in Supplementary Note 8.

### Stimulation of the distal stump to alleviate muscular atrophy

Nerve degeneration after injury occurs via the Wallerian process, with degradation of various components of the axons and myelin sheaths. The timeframe over which these features retain electrical continuity after injury depends on species and the nature of the injured nerve (Supplementary Table 1). For rat models, the EMG response (physiologic action potential) due to distal stimulation disappears after ~36 h, but the protection layer for conduction (myelin) around the nerve remains for 7–10 days. Moreover, the basal lamina remains intact throughout the period of nerve regeneration. In this context, direct electrical stimulation via the distal end of an injured nerve has the potential to offer improved capabilities in preventing denervation atrophy of the innervated muscle tissue. Considering the direction of Wallerian degeneration from the nerve injury site to the distal innervated muscle, the cuff electrode is implanted as distal as possible, in order to trigger effective neuromuscular junction stimulation via a remaining conductive nerve. The rationale for this strategy follows from clinical evidence that neuromuscular electrical stimulation (NMES), via transcutaneous or implantable (i.e., portable) electrodes, has beneficial effects on recovery from denervation injuries, by improving muscle power and function^7,8^, and increasing peripheral blood flow^41^ (Supplementary Note 8).

**Fig. 4:**
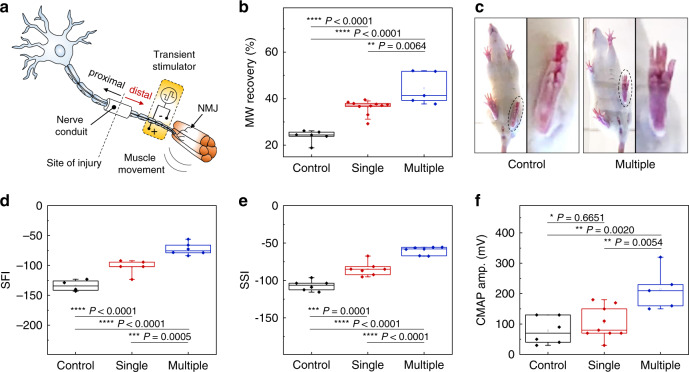
Effect of multiple episodes of electrical stimulation on functional motor recovery 6 weeks after nerve injury. **a** Schematic illustration of the implantation of a wireless electrical stimulator onto the distal stump of a nerve gap model in rats. A bioresorbable nerve conduit (10 mm) is bridged between two ends of the transected sciatic nerve to realize the nerve gap model, and the cuff electrode of the stimulator is implanted on the distal nerve stump. **b** Relative muscle weight (MW) recovery reveals a significant increase in gastrocnemius muscle mass by multiple episodes of distal nerve stimulation. *n* = 5 independent animals per group. **c** Functional gait analysis shows improved function of the injured left hindlimb, with an increase of toe spread in the group with multiple episodes of distal nerve stimulation (circle dotted line). **d**, **e** Dynamic gait analysis further verifies the improved sciatic function index (SFI) and static sciatic index (SSI) in the group with multiple episodes of distal nerve stimulation. *n* = 5 independent animals per group. **f** Electrophysiologic analysis reveals increased amplitude of compound muscle action potential (CMAP), with a significant increase in the group with multiple episodes of distal nerve stimulation. *n* = 5 independent animals per group. The boxplots show the median (center line), the third and first quartiles (upper and lower edge of the box, respectively), and the largest and smallest value that is ≤1.5 times the interquartile range (the limits of the upper and lower whiskers, respectively). Statistical software (Version 6.0) was used for the analysis followed by a t-test and one-way ANOVA with Tukey multiple comparison analysis (*P < 0.05; **P < 0.01; ***P < 0.001; ****P < 0.0001). Data available in source data file.

The following studies focus on these bioresorbable, wireless stimulators interfaced to the distal stumps of the transected sciatic nerves in rat models, with critical nerve gaps of 10 mm bridged by electrospun nanofibrous nerve conduits of poly(l-lactide-co-caprolactone) (Fig. 4a). The cuff electrode is applied right proximal to the trifurcation of sciatic nerve that is secured and insulated by PLGA 75:25 (lactide:glycolide) membrane, to prevent current leakage outside the nerve. The therapy consists of single (20 Hz, 200 μs pulse width, 2–4 V amplitude, 30 min for one-time) or multiple episodes (every other day for 8 days, i.e., five times in total number after injury) of electrical stimulation, designed approximately to mimic physiologic nerve impulses to target muscles, with a goal of maintaining the viability of the muscle fibers and the integrity of the neuromuscular junction (NMJ). Comparisons involve control groups, as defined by animals implanted with non-functional stimulators (no electrical stimulation). Six weeks after sciatic nerve injury, evaluations of functional and electrophysiological recovery determine the extent of therapeutic benefits. Measurement of wet gastrocnemius muscle harvested at 6 weeks reveals significant improvement of relative muscle weight recovery (normalized to contralateral uninjured side) in both single (36 ± 3%) and multiple (44 ± 7%) electrical stimulation groups, as compared to the control group (24 ± 3%) (Fig. 4b). In addition, the multiple stimulation group exhibits much higher recovery of muscle weight compared to the single stimulation group. Gait analysis also shows improved toe spread in the multiple stimulation group, consistent with enhanced sciatic functional recovery (Fig. 4c). The sciatic function indices (SFI) and static sciatic indices (SSI), together with evaluations of functional gait analysis, indicate enhanced functional outcomes after sciatic nerve regeneration. For SFI, the single (−103 ± 12) and multiple stimulation (−73 ± 10) group achieved functional recovery more extensively compared to the control (−134 ± 9) group (Fig. 4d). For SSI, the single (−85 ± 9) and multiple stimulation (−60 ± 5) group show similar benefits compared to the control (−107 ± 7) group (Fig. 4e). Electrophysiological analysis reveals a significant increase in the amplitude of CMAP in the multiple stimulation group (210 ± 70 mV), as compared to the control (80 ± 40 mV) and single (100 ± 50 mV) stimulation groups (Fig. 4f and Supplementary Fig. S21). Taken together, the data support the hypothesis that multiple episodes of distal electrical stimulation have therapeutic benefits on muscle weight regeneration, sciatic functional recovery and electrophysiology and that multiple stimulation episodes result in an accumulative enhancement. We note that stimulators with wired power supply but otherwise similar designs yield results similar to the wireless devices in SFI, SSI, muscle weight and CMAP (Supplementary Fig. S22).

**Fig. 5:**
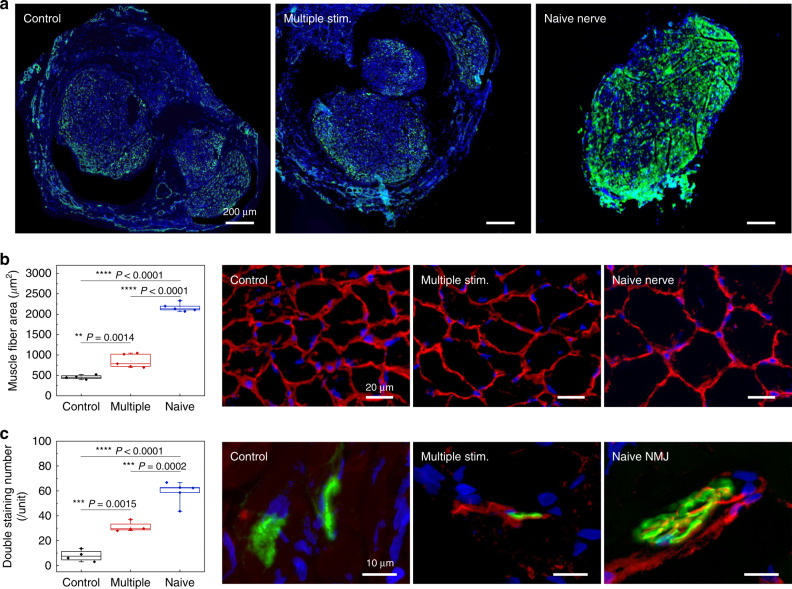
Histological evidence of improved muscular recovery by multiple episodes of distal nerve stimulation 6 weeks after nerve injury. **a** Stained slices show no significant difference in mature axon (Tuj1, green color) signals in the regenerated nerve within the nerve conduit, indicating that the beneficial effect of motor recovery is not via accelerated axon regeneration. *n* = 3 repeated independently with similar results. **b** Immunohistochemical staining of the muscle fiber boundary (laminin, red color) reveals an increased muscle fiber surface area in the group with multiple episodes of distal nerve stimulation. *n* = 4 biologically independent animals. **c** Double staining of the neuromuscular junction demonstrates significantly increased overlapping of pre- (NFM, red color) and postsynaptic (alpha-bungarotoxin, green color) staining for the group with multiple episodes of distal nerve stimulation, indicating an enhanced number of neuromuscular junction (NMJ) and muscle reinnervation. Naive indicates mice with uninjured nerve. *n* = 4 biologically independent animals. The boxplots show the median (center line), the third and first quartiles (upper and lower edge of the box, respectively), and the largest and smallest value that is ≤1.5 times the interquartile range (the limits of the upper and lower whiskers, respectively). Statistical software (Version 6.0) was used for the analysis followed by a t-test and one-way ANOVA with Tukey multiple comparison analysis (*P < 0.05; **P < 0.01; ***P < 0.001; ****P < 0.0001). Data available in source data file.

Histological investigations on the regenerated nerve within the conduit 6 weeks after injury provide information to exclude possible effects of unintentional stimulation of the proximal nerve stump, where stimulation has known beneficial effects on promoting the sprouting of regenerated axons^42^. Immunohistochemical staining reveals that the density of regenerated axons from multiple episodes of the distal nerve stimulation group remains unchanged compared to the control (non-stimulation) group (Fig. 5a, green color for Tuj1-positive staining). These results suggest that the therapeutic outcomes of distal nerve stimulation follow mainly from distal influence. Further studies examine regeneration of the innervated gastrocnemius muscle at week 6. As shown in Fig. 5b, immunofluorescent staining of the muscle fiber boundary (laminin, red color) reveals a significant increase in the surface area of the regenerated muscle fibers in the multiple stimulation group compared to the control (non-stimulation) group. Furthermore, immunofluorescent staining of the innervated gastrocnemius muscle neuromuscular junction (NMJ) shows that multiple episodes of distal stimulation lead to a higher number of functional NMJs (co-localization of neurofilament in red color and alpha-Bungarotoxin in green color, Fig. 5c), in comparison with the control (non-stimulation) group. Collectively, these findings suggest that the multiple episodes of distal nerve stimulation result in enhancement of neuromuscular reinnervation, without an acceleration of axon regeneration.

**Fig. 6:**
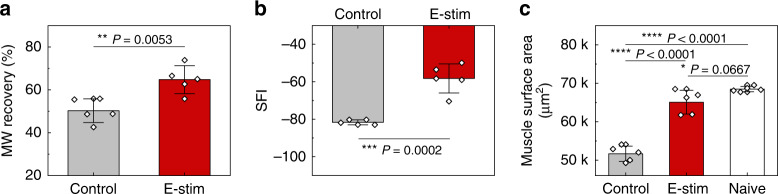
Early protective mechanism by a single episode of distal nerve stimulation 12 days after denervation injury. **a** Relative muscle weight (MW) reveals a significant preservation of gastrocnemius muscle mass following a single episode of distal nerve stimulation. **b** Dynamic gait analysis reveals that the sciatic function index (SFI) is significantly preserved by this single stimulation. **c** Hematoxylin and Eosin (H&E) staining of the affected gastrocnemius muscle fiber reveals an improved maintenance of muscle fiber surface area by single stimulation. In **a**, **b**, and **c** the results are shown as means ± s.e.m (*n* = 5 biologically independent animals per groups). Statistical software (Version 6.0) was used for analysis followed by a *t*-test and one-way ANOVA with Tukey multiple comparison analysis (*P < 0.05; **P < 0.01; ***P < 0.001; ****P < 0.0001). Data available in source data file.

Denervated muscle fibers exhibit spontaneous, recurring single muscle fiber discharges detectable by single fiber electromyography^43^. Application of a single episode of electrical stimulus on the distal stump, therefore, provides an additional opportunity to investigate the mechanism of direct nerve stimulation via the implantable electrode on the distal nerve stump. The relative gastrocnemius muscle weight at 12 days reveals significant preservation of muscle weight ratio in the stimulation group (65 ± 7.5%) compared to the control (non-stimulation) group (50 ± 8.1%) (Fig. 6a). Gait function analysis demonstrates the preservation of SFI in the stimulation group (−58 ± 7.8), as compared to the control (non-stimulation) group (−82 ± 1.3) at 12 days (Fig. 6b). Gastrocnemius muscle fiber histological analysis at the same time point reveals a delayed decrease in muscle fiber surface area (65k ± 3k µm^2^) in the stimulation group when comparing to the control (non-stimulation) group (52k ± 2k µm^2^) (Fig. 6c). Taken together, the electrical stimulation delivered from the distal nerve stump to the target muscle contributes to preservation of innervated muscle from denervation, atrophy and functional loss in the early phase of recovery, thus providing more receptive muscle fiber for subsequent reinnervation by motor neuron regeneration at later phase.

In conclusion, the materials, device architectures, and fabrication strategies introduced here serve as the foundations for long-lived, stretchable, and wireless bioresorbable electrical stimulation platforms designed to enhance neuromuscular reinnervation after denervation injury. A combined set of in vitro and in vivo studies show that bioresorbable polyurethane materials that incorporate dynamic covalent networks serve critical roles as substrates and encapsulation layers, to support electrical reliability, mechanical stability, bioresorbability, and biocompatibility. These materials and device concepts may be useful for other classes of bioresorbable electronic implants, such as those in electrical stimulators for bone healing, spinal cord and brain therapy, and cardiac pacing. Evaluations on rat models of peripheral nerve injury suggest that this paradigm for neuromuscular electrical stimulation on the distal nerve stump maintains muscle receptivity and preserves functional abilities for recovery, via impeding the muscle denervation atrophy. This stimulation strategy may be extended to dual electrical stimulation on both proximal and distal nerve stumps to generate synergistic effects of neuroregeneration and functional muscle recovery.

## Methods

### Synthesis of polycaprolactone-based polyurethane (b-DCPU)

Polycaprolactone (PCL) triol (PCL-triol; average *M*_n_ ~900 g/mol), hexamethylene diisocyanate (HDI), anhydrous butyl acetate, and tin(II) 2-ethylhexanoate (Sn(Oct)_2_) were obtained from Sigma-Aldrich. Supplementary Fig. S2 presents the molecular structures of the chemical precursors (PCL-triol, HDI) and that of the final cross-linked polymer network. The crosslinking density depends on the molar ratio of HDI to PCL-triol. The synthesis began with melting 2.7 g of PCL-triol in a dried glass vial at 60 °C, followed by mixing HDI (504, 576, 648, and 720 μL for b-DCPU 70, 80, 90 and 100, respectively) and butyl acetate (15 mL) until full dissolution at 60 °C. Cooling this solution and adding Sn(Oct)_2_ as a biocompatible catalyst (6.69 μL)^38^, followed by drop-casting on a hydrophobic surface prepared by immersion of a Si wafer into 0.2 vol.% trichloro(octadecyl)silane (Sigma-Aldrich, USA) in hexane yielded films with thickness defined by the volume and area. Substrates were placed for 24 h in an oven at 60 °C for solvent evaporation and curing, resulting in semi-transparent polymer films. (Detailed property characterization of b-DCPU is described in Supplementary Note 3) To remove potentially cytotoxic residual reagents, we immersed the resulting b-DCPU films in PBS (pH 7.4) at 80 °C for 2 days and thoroughly washed the sample using deionized water. We used the as-made b-DCPU films for degradation tests.

### Preparation of bioresorbable components and assembly of wireless nerve stimulator

Laser-cut metal coils (~50 μm thick Mg or ~15 μm thick Mo; Goodfellow, USA) transferred onto substrates of b-DCPU served as the receiving antennas for RF power harvesting. Solid-state diffusion of boron (tube furnace at 1050 °C with N_2_ flow) and phosphorus (tube furnace at 1050 °C with N_2_ flow) through a photolithographically defined hard mask of SiO_2_ formed by plasma-enhanced chemical vapor deposition (PECVD) yielded a PIN diode with a monocrystalline membrane of silicon derived from a silicon-on-insulator (SOI) wafer (top silicon 1.2 μm thick, p-type; SOITEC, France). Removing the buried oxide by immersion in hydrofluoric acid allowed release and transfer printing of the membranes onto a sacrificial layer of diluted poly(pyromellitic dianhydride-co-4,4′-oxydianiline) (D-PI; ~200 nm) on a film of poly(methyl methacrylate) (PMMA; ~300 nm) on a silicon wafer. Photolithographic patterning and reactive ion etching (RIE) determined the lateral dimensions of the doped Si for integration into diodes. Spin-casting an overcoat of D-PI and dry etching through the underlying D-PI and PMMA defined an open mesh layout. Immersion in acetone released the PIN diode and allowed its transfer to the PLGA substrate (~30 μm). Oxygen RIE removed the D-PI layers during/after the transfer printing. Laser-cutting of Mo foil (~15 μm thick) (or Mg foil, 50 μm thick) into 200-μm wide strips yielded the electrical extension electrodes and interconnects to the cuff for the nerve interface. These bioresorbable components (RF coil, PIN diode and extension electrode) were collected on a b-DCPU substrate and electrically interconnected with a bioresorbable conductive wax paste^44^. For strong adhesion of b-DCPU to itself and to other bioresorbable materials, the bioresorbable components on b-DCPU substrate were pre-treated by oxygen plasma (200 mtorr, 200 W, 120 s; Reactive Ion Etch Plasma System, Nordson March, CA, USA). Covering the components with b-DCPU and hot pressing the entire system yielded a compact, double-coil structure with openings for interconnects.

### Mechanical test setup

Tensile testing was conducted by Sintech 20 G (MTS Systems, MN, USA) with a stretching rate of 15 mm min^−1^ on dog-bone polymer specimens (length: 22 mm; width: 5 mm; ASTM Standard D1708). The slopes of stress–strain curves within the elastic range defined the Young’s modulus values. Fatigue tensile testing (RSA G2 Dynamic Mechanical Analyzer, TA Instruments, IL, USA) used similar specimens. 180-degree peeling tests (ASTM F2256) enabled measurements of adhesion between two layers of b-DCPU joined using a hot pressing method. Evaluation of adhesion between b-DCPU (2.5 cm × 1 cm × 200 μm) and other materials (2.5 cm × 1 cm) used plasma-treated materials grown (SiO_2_; PECVD) or anchored (Mg, Mo; super glue) on Si wafers. Peeling the b-DCPU layer at 90 degrees from these materials yielded the adhesion results. In all cases, a layer of PLA (thickness: 50 µm) bonded to the backside of b-DCPU eliminated the energy dissipation from an elastic deformation. The stretching rates were 60 mm min^−1^. Twice the plateau value of the tensile force divided by the width of these samples defined the adhesion energy^45^. Rheological behaviors determined with a modular compact rheometer (Anton Parr MCR302, IL, USA; 25-mm-diameter cone plate (CP25); amplitude at 5%) defined changes in mechanical properties during degradation.

### Tests of water permeation

A Mg resistor structure formed the basis of tests of water permeation properties of candidate materials for encapsulation. Photolithography and lift-off using a positive photoresist (AZ 4620, MicroChemicals) yielded patterns of Mg (100–500 nm) on a Si wafer with a layer of SiO_2_ on its surface. A layer of Cr/Au (10/100 nm) patterned in the same manner defined pads for electrical probing. Placing a layer of encapsulation (~300 µm thick) on the Mg resistor and sealing a well structure formed in PDMS (Sylgard 184, Dow Corning, USA) and filled with PBS (pH 7.4) yielded a testing platform. DI water was added periodically to maintain the concentration of PBS due to natural evaporation. The sample was placed in an oven at 37 °C, and the resistance values of samples were collected every hour and averaged from five different samples per group. Additional evaluations defined the time-dependent changes in the resistance of Mo traces (~15 µm thick) without encapsulation, encapsulated with b-DCPU 80 (~100 µm thick), and encapsulated with PLGA (65:35 (lactide:glycolide); *M*_w_ 40–75 K; ~100 µm thick), after immersion in PBS (pH 7.4; 37 °C).

### Modeling of reactive diffusion for polymer encapsulated Mg

A one-dimensional analytical model can be used to describe the reaction and diffusion processes of a single Mg layer and polymer (PLGA or b-DCPU) encapsulated Mg while immersed in an aqueous solution (PBS at pH 7.4, 37 °C)^44,46^. The model applies since the single Mg layer thickness (*h*_0_) and the polymer/Mg specimen thickness (*h*_0_ + *h*_polymer_), where *h*_0_ and *h*_polymer_ are the initial thicknesses of the Mg layer and polymer encapsulation, respectively, are much smaller compared to their diameters (Figure in Supplementary Note 10). The resistance of Mg is given by $R = \frac{{R_0h_0}}{h}$ where *R*_0_ is the initial resistance. Using the equation for normalized Mg thickness (Eq.12 in Supplementary Note 10), the critical time *t*_*c*_ (i.e., functional lifetime) can be determined for the resistance to reach a critical value (i.e., *R* = 500 Ω) by using *R* ~ 87 Ω, measured from experiments, and setting $\frac{{h\left( {t_c} \right)}}{{h_0}} = 0.175$. The material parameters used in the polymer encapsulation model are given in references^44,46^ as *M*_Mg_ = 24 g/mol, *M*_H2O_ = 18 g/mol, *ρ*_Mg_ = 1.738 g/cm, *w*_0_ = 1 g/cm, *k*_Mg_ = 1.2 × 10^−3^/s, and *D*_Mg_ = 6.0 × 10^−16^ m^2^/s. The calculated diffusivities *D*_polymer_ of PLGA and b-DCPU are reported in Supplementary Note 5.

### Test of polymer swelling and dissolution in biofluids

Tests used square samples (~15 mm × ~15 mm) of PLGA, b-DCPU, and PEG containing b-DCPU with various thickness (300–500 µm) immersed into bath of 0.1 M phosphate-buffered saline (PBS, Sigma-Aldrich, USA) at temperatures of 23, 37, 60 and 80 °C. Removing the samples at designated time intervals, rinsing them with deionized (DI) water and drying the residual water under vacuum for one day prepared the samples for weighing. Experiments were terminated when the b-DCPU changed from a solid film to a gel-like substance as a result of the degradation processes. Comparison of the weights before and after drying under the vacuum yielded the water uptake.

### Synthesis of polycaprolactone/poly(ethylene glycol)-based polyurethane

Poly(ethylene glycol) (PEG) (average *M*_n_ ~  2000 g/mol) was obtained from Sigma-Aldrich. All reagents were used as received. By varying the ratio of PCL-triol to PEG, different types of PEG containing b-DCPU films were fabricated as referred by the weight fraction of PCL-triol and PEG based on b-DCPU 80 (e.g., PCL22-PEG58-U = 22 wt% PCL-triol and 58 wt% PEG). The mixture was melted at 60 °C, and then ~15 mL of butyl acetate was added, until full dissolution of the mixture occurred at 60 °C. After cooling, 6.69 µL of the Sn(Oct)_2_ was added, and the resulting liquid was drop-cast on a dry, pristine glass slide. Substrates were placed for 24 h in an oven at 60 °C for solvent evaporation and curing, resulting in semi-transparent polymer films.

### In vitro biocompatibility test

A mouse fibroblast cell line was purchased from ATTC (ATCC^®^ CCL-1^™^) along with their media (ATCC^®^ 30-2003^™^). The cells were maintained and cultured in 25-cm^2^ flasks according to the manufacturer’s protocols. Cells were used for viability assay after the first or second subculture. Three different b-DCPU samples (~0.5 mm × ~0.5 mm) were prepared: (1) normal b-DCPU (Pristine); (2) degraded b-DCPU with gel-type texture (Deg.) degraded in PBS at 95 °C for 1 month; (3) fully dissolved b-DCPU solution (Sol.) degraded in PBS at 95 °C for 2 months. It must be noted that Pristine does not indicate as-made b-DCPU sample. As we aforementioned, Pristine sample was fabricated by immersing b-DCPU in PBS (pH 7.4) at 80 °C for 2 days and thoroughly washing the sample using deionized water to remove potentially cytotoxic residual reagents. All samples were sterilized by EtO gas sterilizer (Anprolene^®^ AN74i) then was exposed to ultraviolet (UV) light for at least 30 min. After placing the sample into the tissue culture plate (Costar-3548), cells were seeded and maintained on the sample at a concentration of 10,000 cells/mL. After 24 h, resazurin assay^47^ was performed. Resazurin sodium salt was purchased from Sigma-Aldrich (R7017) and fluorescence was measured with Cytation5 (Biotek^®^). For live and dead staining, cells were cultured and maintained in the same condition for 3 days. Live/dead viability/cytotoxicity Kit (L3224) was purchased from Invitrogen™, and cells were stained according to the manufacturer’s protocols.

### In vivo biocompatibility studies

Female CD1(8 weeks old) mice were purchased from Charles River Laboratories. All procedures were approved by the Institutional Animal Care and Use Committee of Northwestern University (protocol IS00005877). The mice were anesthetized with isoflurane gas (~2%), HDPE and samples of b-DCPU were implanted subcutaneously through dorsal incision. Following 14 or 28 days of implantation, the mice were sacrificed for histology. Tissue samples were fixed in 10% neutral buffered formalin, embedded in paraffin, sectioned and stained with Hematoxylin and Eosin (H&E) for histological analysis. Polymorphonuclear cells and lymphocytes were identified by morphology from at least three distinct regions by 400x fields per samples. Histological scores were assessed as reported earlier. Five random locations were chosen for the capsule thickness measurements from optical micrographs at ×10 magnification.

### In vivo stability of b-DCPU and stimulation of normal (uninjured) sciatic nerves in rat models

Following anesthetization, a biodegradable stimulator was implanted and interfaced to the normal (i.e., uninjured) sciatic nerve 5 mm proximal to the trifurcation. The surgical site was then closed as described above. Implanted stimulators were wirelessly activated in order to deliver therapeutic electrical stimulation (monophasic, 200 μs pulse, 20 Hz frequency, minimum amplitude over threshold) to the normal nerve for half hour per day.

### Simulations of mechanical characteristics

The finite element analysis (FEA) commercial software ABAQUS (Analysis User’s Manual 2016) was utilized to optimize the interconnect layout and to study the corresponding mechanical characteristics. The objective was to decrease the strain level in Mo wires in the interconnect under different typical loads. The b-DCPU was modeled by hexahedron elements (C3D8R) while the thin Mo layer (15 µm thick) was modeled by shell elements (S4R). The minimal element size was 1/8 of the width of the Mo wires (20 µm), which ensured the convergence, and the accuracy of the simulation results. The elastic modulus (*E*) and Poisson’s ratio (*ν*) used in the analysis were *E*_Mo_ = 330 GPa, *ν*_Mo_ = 0.29, *E*_PU_ = 0.8 MPa, *ν*_PU_ = 0.5.

### Simulation of the electromagnetic characteristics

The commercial software ANSYS HFSS was used to perform electromagnetic (EM) finite element analysis and determine the inductance, Q factor, and scattering parameters (Supplementary Fig. S2) of the bioresorbable, implantable wireless stimulator (2 layers of 17 turns each, 12 mm diameter). Lumped ports were used to obtain the scattering parameters *S*_nm_ (Supplementary Fig. S2a, b) and port impedance *Z*_nm_ (Fig. S2c) the coils. An adaptive mesh (tetrahedron elements) and a spherical radiation boundary (radius of 1000 mm) were adopted to ensure computational accuracy. The frequency-dependent impedance of the resonant system is given by

1 $$Z(\omega ) = \frac{{R + j\omega L}}{{1 - \omega ^2LC + j\omega LC}},$$

where, *ω* is the angular frequency, *L* is the inductance, *R* is the resistance, and *C* is the parasitic capacitance. The quality factor (*Q*) was obtained from the |Z| curve in Supplementary Fig. S2d as $Q_n = \frac{{\left( f \right)}}{{\left( {{\Delta} f} \right)}} = 23.8$, where *f*, and Δ*f* represent the working frequency, and 3 dB bandwidth, respectively. The resistance *R*_0_ and inductance *L*_0_ at resonance are calculated as $R_0 = \frac{{Z_0}}{{1 + Q^2}} = 40.5\,\,{\Omega}$ and $L_0 = \frac{{QR_0}}{\omega } = 10.58\,{\mathrm{\mu}} {\mathrm{H}}$, where *Z*_0_ is the impedance at resonance. The parasitic capacitance, considered constant in the absence of changing electrical permittivity around the coil core, is $= \frac{1}{{\left( \omega \right)^2L_0}}\frac{{Q^2}}{{1 + Q^2}} = 11.35\,{\mathrm{pF}}$^48^. The frequency-dependent inductance (Supplementary Fig. S2e) was calculated as

2 $$\omega L(\omega ) = \frac{{Z_i(\omega ) + \omega C|Z(\omega )|^2}}{{[1 + \omega CZ_i(\omega )]^2 + [\omega CZ_r(\omega )]^2}},$$

where *Z*_*i*_ and *Z*_*r*_ are the imaginary and real parts of the impeadance (Supplementary Fig. S2c), respectively. The power transfer efficiency *η* was calculated as

3 $$\eta = |S_{21}|^2 \times 100\%,$$

for different values of load resistance (Supplementary Fig. S1d).

At the self-resonance frequency, the parasitic capacitance dominates the behavior of the wireless stimulator, making it an open circuit. The voltage between the device then represents the full voltage value since there is no additional circuit load sharing the voltage in the device. The wireless devices reported here operate at a frequency that is more than three times higher than that of previously published devices^15^. According to Faraday’s law of induction, the output voltage can be determined by the rate of change of the magnetic flux. Therefore, operating the system at a higher frequency (still below the threshold when electromagnetic tissue absorption becomes relevant) will increase the output of the wireless stimulation sensor, thereby allowing for a reduction in the size and number of turns in the coils for a specific voltage output.

### In vivo implantation of wireless, bioresorbable stimulators and protocol for distal nerve stimulation

Studies of in vivo therapeutic effects relied on a critical nerve gap model of the rodent sciatic nerve. The experimental procedures with animals were approved by the ACUC committee at UCLA (protocol number 2016-101-03) and were carried out according to the institutional guidelines. Adult female Sprague-Dowley rats (Charles River Laboratories) weighing 200–250 g were used in the following experiments, and six animals were used in each group. Three experimental groups were included: (1) No electrical stimulation (control group), (2) single electrical stimulation (single group), and (3) multiple electrical stimulation (multiple group). All groups of animals received the following procedures of nerve gap injury, bridged with electrospun nanofibrous nerve conduit and implanted with an electrical stimulator. An incision of the skin and gluteal muscle of the left hindlimb revealed the sciatic nerve. A critical nerve gap of 10 mm was created 5 mm above the trifurcation of the sciatic nerve, repaired by electrospun nanofibrous conduit with 8-0 Nylon monofilament sutures. The cuff electrode was placed at the distal stump of nerve, wrapped and secured with a sheet of PLGA 75:25 (lactide:glycolide) membrane for insulation. The bioresorbable receiver antenna was implanted into adjacent subcutaneous layers for wireless stimulation. After the implantation, the overlying muscle layers and skin were sutured with 4–0 absorbable sutures to close the surgery site. Immediate wireless stimulation was delivered through radiofrequency communication with a transmission powered by a waveform generator. Implanted stimulators were wirelessly activated to deliver electrical stimulation (monophasic, square waveform, 200 μs pulse width, 20 Hz frequency, minimum amplitude over threshold, 2–4 V) to the injured nerve for 30 min for the single stimulation group. For the multiple stimulation group, the stimulation was delivered for four additional episodes on 2, 4, 6, and 8 consecutive days postoperatively.

### MicroCT imaging

Rats were imaged *post mortem* with a preclinical microCT imaging system (nanoScan PET/CT, Mediso-USA, Boston, MA). Data acquisition used 2.2x magnification, <60 µm focal spot, 1 × 4 binning, with 720 projection views over a full circle, using 70 kVp/520 µA, with a 300 ms exposure time. The projection data was reconstructed with a voxel size of 68 µm and using filtered (Butterworth filter) backprojection software from Mediso. The reconstructed data was visualized and segmented in Amira 2019.2 (FEI, Houston, TX). A non-local-means filter was used to reduce image artifacts.

### Fabrication of nerve conduits

An electrospinning technique produced nanofibrous nerve conduits^49^. In brief, nonwoven aligned nanofibrous nerve conduits composed of poly(l-lactide-co-caprolactone) (70:30, Purac Biomaterials), poly(propylene glycol) (Acros Organics) and sodium acetate (Sigma) were fabricated by using a customized electrospinning process. To make tubular scaffolds with aligned nanofibers in the longitudinal direction on luminal surface, a rotating mandrel assembly with two electrically conductive ends and a central non-conductive section was used. The jet stream of polymer solution from the spinneret whipped between the two conductive ends, resulting in longitudinally aligned nanofibers forming a tubular scaffold on the non-conductive portion of the mandrel. To enhance the mechanical strength of the scaffolds, outer layers of random nanofibers were deposited on this layer of longitudinally aligned fibers^50^.

### Electrophysiology measurements

The rat sciatic nerve was re-exposed and dissected freely from surrounding soft tissue. Electrical stimuli (single-pulse shocks, 1 mA, 0.1 ms) were applied to the intact sciatic nerve trunk at the point 5 mm proximal to the conduit suturing point^51^. The amplitudes of compound muscle action potentials (CMAPs) were recorded on the innervated gastrocnemius muscle from 1 V to 12 V or until a supramaximal CMAP was reached. Normal CMAPs from the non-injured right side of sciatic nerve were also recorded for normalization. Grass Tech S88X Stimulator (Astro-Med Inc.) was used for the test and PolyVIWE16 data acquisition software (Version 1.1, Astro-Med, Inc.) was used for recording. Recovery rate was the ratio of amplitude of injured hindlimb’s CMAP to contralateral normal hindlimb’s CMAP.

### Gait function analysis

These studies used a walking track equipped with a video-based system, modified from that used in a previous study^52^. The apparatus consisted of a Plexiglas chamber that was 80 cm long, 6 cm wide, and 12 cm high with transparent glass underneath the walking track. The footprint was recorded using an EX-F1 digital camera (Casio, Tokyo, Japan) from below the walking track. The task was repeated until 5 or 6 satisfactory walks of at least 4 steps without pause were obtained. In this study, only the hindlimb stepping patterns were analyzed. The digital images obtained from each trial were processed with a threshold setting to detect the boundary of the soles, and critical points for determining the derivation of paw indices were determined using Matlab software (MathWorks, Natick, MA, USA). After identifying sequential footprints, sciatic function index^53^ (SFI) and static sciatic index^54^ (SSI) were calculated. Each parameter was averaged from at least 20 footsteps.

### Histological analysis and immunostaining

The nerve conduits and muscle were harvested and fixed in 4% paraformaldehyde at 4 °C for 2 h. After washing with PBS, tissues were cryoprotected with 30% sucrose in PBS at 4 °C overnight, and were then embedded in optimum cutting temperature (OCT) compound and were frozen at −80 °C. The frozen samples were cryosectioned longitudinally and transversely with the thickness of 10 μm at −20 °C. The slices were placed onto Super frost plus slides and stored in −20 °C. Immunostaining was performed for histological analysis. Slices were permeabilized with 0.5% Triton X-100 in PBS for 30 min, blocked with 5% Normal Donkey Serum (NDS) in PBS for 1 h, and then samples were incubated with primary antibody against Tuj1 (1:1000, Abcam, ab18207) in 4 °C overnight. Slides were washed three times for 15 min in PBS and incubate with corresponding A AlexaFluor 488 goat anti-rabbit secondary antibodies (1:400, Thermofisher scientific, A32731) in PBS for 1 h in room temperature. Finally, samples were cultured with DAPI(1:1000, Sigma, 28718-90-3) for 5 min. After further PBS washing, coverslips were mounted and viewed with a fluorescent microscope (Zeiss).

Muscles sections were collected on microscope slides and fixed by 4% PFA for 10 min and then washed by 1x PBS three times and penetrated by 0.5% triton X-100 in PBS for 30 min. Samples were incubated at room temperature for 2 h with 5% NDS for blocking. Afterward, samples were incubated with primary antibody against laminin (1:300, Sigma-Aldrich, L9393) or neurofilament (1:100, Sigma-Aldrich, N4142) in 4 °C overnight. Laminin and neurofilament antibody was diluted in 1% NDS and PBS, respectively. The following day, samples were washed three times for 15 min in PBS and incubated with corresponding AlexaFluor 546 secondary antibody (1:300, Donkey anti-rabbit, Life Technologies Corporation, A10040) and alpha-BTX (1:100, Thermo Fisher Scientific Company, B13422) in PBS for 1 h in room temperature. Finally, samples were cultured with DAPI for 5 min. Between these steps, slides were washed with PBS three times for 15 min to remove unbonded molecules or antibodies. Samples were mounted by fluormount media and sealed by microscope cover glass. The slides were then put in 4 °C refrigerator overnight to allow the cover glass to set before imaging. A Zeiss microscope was used to image laminin and NMJ staining. Both laminin and neurofilament were labeled with red and NMJ was labeled in green (alpha-BTX). Z-stack was collected for the NMJ-neurofilament image to show the connections between them. ImageJ was used to measure the area of muscle fiber and to project the Z-stacks to a single image (image > stacks > Z-project > Max intensity). The image obtained from this process was used to count NMJ in sections.

### Statistical analysis

Results are reported as mean ± 6 SD, unless otherwise noted. Statistical analyses were performed using Statistical software (Version 6.0, Statsoft, Tulsa, Oklahoma) followed by a *t*-test and one-way ANOVA (with Tukey multiple comparison analysis for the difference between groups). *P < 0.05, *P < 0.01, ***P < 0.001.

### Reporting summary

Further information on experimental design is available in the Nature Research Reporting Summary linked to this article.

## Supplementary information

Supplementary Information

Reporting Summary

## Data Availability

Source data are provided with this paper.

## Source data

Source Data
