# Author Correction: Stretchable, dynamic covalent polymers for soft, long-lived bioresorbable electronic stimulators designed to facilitate neuromuscular regeneration

**DOI:** 10.1038/s41467-020-20857-y

**Published:** 2021-01-12

**Authors:** Yeon Sik Choi, Yuan-Yu Hsueh, Jahyun Koo, Quansan Yang, Raudel Avila, Buwei Hu, Zhaoqian Xie, Geumbee Lee, Zheng Ning, Claire Liu, Yameng Xu, Young Joong Lee, Weikang Zhao, Jun Fang, Yujun Deng, Seung Min Lee, Abraham Vázquez-Guardado, Iwona Stepien, Ying Yan, Joseph W. Song, Chad Haney, Yong Suk Oh, Wentai Liu, Hong-Joon Yoon, Anthony Banks, Matthew R. MacEwan, Guillermo A. Ameer, Wilson Z. Ray, Yonggang Huang, Tao Xie, Colin K. Franz, Song Li, John A. Rogers

**Affiliations:** 1 Center for Bio-Integrated Electronics, Northwestern University, Evanston, IL 60208 USA; 2 Querrey Simpson Institute for Biotechnology, Northwestern University, Evanston, IL 60208 USA; 3 Department of Materials Science and Engineering, Northwestern University, Evanston, IL 60208 USA; 4 Department of Bioengineering, University of California, Los Angeles, Los Angeles, CA 90095 USA; 5 Division of Plastic and Reconstructive Surgery, Department of Surgery, National Cheng Kung University Hospital, College of Medicine, National Cheng Kung University, Tainan, 70456 Taiwan; 6 International Research Center for Wound Repair and Regeneration, National Cheng Kung University, Tainan, 70456 Taiwan; 7 School of Biomedical Engineering, Korea University, Seoul, 02841 Republic of Korea; 8 Interdisciplinary Program in Precision Public Health, Korea University, Seoul, 02841 Republic of Korea; 9 Department of Mechanical Engineering, Northwestern University, Evanston, IL 60208 USA; 10 Department of Medicine, University of California, Los Angeles, Los Angeles, CA 90095 USA; 11 State Key Laboratory of Structural Analysis for Industrial Equipment, Dalian, University of Technology, 116024 Dalian, China; 12 Department of Engineering Mechanics, Dalian University of Technology, 116024 Dalian, China; 13 International Research Center for Computational Mechanics, Dalian University of Technology, 116024 Dalian, China; 14 State Key Laboratory of Chemical Engineering, College of Chemical and Biological Engineering, Zhejiang University, 310027 Hangzhou, China; 15 Department of Biomedical Engineering, Northwestern University, Evanston, IL 60208 USA; 16 State Key Laboratory of Mechanical System and Vibration, Shanghai Jiao Tong University, 200240 Shanghai, China; 17 Center for Developmental Therapeutics, Chemistry Life Processes Institute, Northwestern University, Evanston, IL 60208 USA; 18 Department of Neurological Surgery, Washington University School of Medicine, St. Louis, MO 63110 USA; 19 Center for Advanced Molecular Imaging, Northwestern University, Evanston, IL 60208 USA; 20 School of Advanced Materials Science and Engineering, Sungkyunkwan University (SKKU), Suwon, 16419 Republic of Korea; 21 Center for Advanced Regenerative Engineering, Northwestern University, Evanston, IL 60208 USA; 22 Department of Surgery, Feinberg School of Medicine, Northwestern University, Chicago, IL 60611 USA; 23 Department of Civil and Environmental Engineering, Northwestern University, Evanston, IL 60208 USA; 24 Regenerative Neurorehabilitation Laboratory, Biologics, Shirley Ryan AbilityLab, Chicago, IL 60611 USA; 25 Department of Physical Medicine and Rehabilitation, Feinberg School of Medicine, Northwestern University, Chicago, IL 60611 USA; 26 The Ken & Ruth Davee Department of Neurology, Feinberg School of Medicine, Northwestern University, Chicago, IL 60611 USA; 27 Department of Neurological Surgery, Feinberg School of Medicine, Northwestern University, Chicago, IL 60611 USA

**Keywords:** Biomedical materials, Implants, Electronic devices

Correction to: *Nature Communications* 10.1038/s41467-020-19660-6, published online 25 November 2020

The original version of this Article contained an error in the spelling of the author Hong-Joon Yoon, which was incorrectly given as Hong-Joon Yun. This has now been corrected in both the PDF and HTML versions of the Article.

