# Supplementary material for: Stretchable, dynamic covalent polymers for soft, long-lived bioresorbable electronic stimulators designed to facilitate neuromuscular regeneration
Source: Nat Commun. 2020 Nov 25;11:5990. doi: 10.1038/s41467-020-19660-6 (PMC7688647; doi:10.1038/s41467-020-19660-6)
Supplement: Supplementary file 1 — Supplementary Information [file 41467_2020_19660_MOESM1_ESM.pdf]

Supplementary Information

Stretchable, dynamic covalent polymers for soft, long-lived bioresorbable electronic stimulators designed to facilitate neuromuscular regeneration

Yeon Sik Choi, Yuan-Yu Hsueh, Jahyun Koo, Quansan Yang, Raudel Avila, Buwei Hu, Zhaoqian Xie, Geumbee Lee, Zheng Ning, Claire Liu, Yameng Xu, Young Joong Lee, Weikang Zhao, Jun Fang, Yujun Deng, Seung Min Lee, Abraham Vázquez-Guardado, Iwona Stepien, Ying Yan, Joseph W. Song, Chad Haney, Yong Suk Oh, Wentai Liu, Hong-Joon Yun, Anthony Banks, Matthew R. MacEwan, Guillermo A. Ameer, Wilson Z. Ray, Yonggang Huang, Tao Xie, Colin K. Franz, Song Li, John A. Rogers

Table of Contents

Supplementary Note 1. Electrical performance characterization of the wireless power transfer system 3

Supplementary Note 2. Limitation of existing bioresorbable encapsulation materials 4

Supplementary Note 3. Characterization of b-DCPU 5

Supplementary Note 4. Adhesion mechanisms with b-DCPU 6

Supplementary Note 5. Permeation behavior of b-DCPU 7

Supplementary Note 6. Mechanical stretchability of b-DCPU 8

Supplementary Note 7. Degradation behavior of b-DCPU 9

Supplementary Note 8. Biocompatibility and toxicity of b-DCPU 10

Supplementary Note 9. Conventional neuromuscular electrical stimulation systems 11

Supplementary Note 10. Modeling of reactive diffusion for polymer encapsulated Mg 12

Supplementary Table 1. The alive response after injury at lower limb nerve 15

Supplementary Fig. 1 | Electrical performance characteristics of the wireless power transfer system. 16

Supplementary Fig. 2 | Electrical characteristics of the transient inductor. 18

Supplementary Fig. 3 | Chemistry associated with the synthesis of b-DCPU. 19

Supplementary Fig. 4 | Characteristics of b-DCPU. 20

Supplementary Fig. 5 | Schematic illustrations of bonding mechanisms associated with b-DCPU. 21

Supplementary Fig. 6 | Schematic illustration of the thermally activated catalytic bond exchange reaction 22

Supplementary Fig. 7 | Water permeation properties of b-DCPU and PLGA. 23

Supplementary Fig. 8 | Design optimization for serpentine-shaped electrodes based on FEA 24

Supplementary Fig. 9 | Mechanical stretchability of b-DCPU encapsulated Mo serpentine electrodes. 25

Supplementary Fig. 10 | Mechanical and optical properties of bioresorbable polyurethane. 26

Supplementary Fig. 11 | Swelling properties of PLGA and b-DCPU. 27

Supplementary Fig. 12 | Bioresorbable behavior of b-DCPU 80 - rheological performance and changes in mechanical properties. 28

Supplementary Fig. 13 | Dissolution kinetics of bioresorbable encapsulation.................................... 29

Supplementary Fig. 14 | Mechanical reliability of the bioresorbable electrical stimulator.................. 30

Supplementary Fig. 15 | Three-dimensional computed tomography images of a mouse collected 1 week after the implantation of a bioresorbable electrical stimulator. 31

Supplementary Fig. 16 | Photographs of three different designs for the Mo electrodes. 32

Supplementary Fig. 17 | Photographs of bioresorbable electrical stimulators with different designs for the extension electrodes. 33

Supplementary Fig. 18 | Photographs of cuff degradation. 34

Supplementary Fig. 19 | *In vivo* evaluations of biocompatibility of b-DCPU encapsulation. 35

Supplementary Fig. 20 | In vivo biocompatibility evaluations of b-DCPU. 36

Supplementary Fig. 21 | Electrophysiologic analysis of re-innervated gastrocnemius muscle at 6 weeks. 38

Supplementary Fig. 22 | Comparison studies of the effect of wired and wireless chronic electrical stimulation on functional recovery. 39

Supplementary Fig. 23 | Schematic illustration of double-layer reactive diffusion with organic encapsulation... 40

Reference 41

Supplementary Note 1. Electrical performance characterization of the wireless power transfer system

Schematic illustration of the wireless power transfer systems (Fig. S1a) shows the input, transmitting, and output voltage (or power). "Input" indicates the programmed voltage and frequency at the waveform generator. "Transmitting" corresponds to the transmitting voltage (or power) at the primary coil (i.e. transmission (Tx) coil). Depending on the circuit structure, the transmission voltage (or power) is the same or smaller than the input voltage (or power). The input and transmission frequencies are the same. "Output" corresponds to the applied voltage (or power) at the nerve with internal resistance (R_{Load}). Monophasic electrical pulses (alternative current (AC) with programmed duration) are generated by a waveform generator. The voltage can be further amplified with an amplifier. The generated waveforms pass to the Tx coil. This RF power (i.e. transmission power) wirelessly transfers to the implanted bioresorbable receiver (Rx) coil and rectifying circuit, including the RF diode. When the transmitting frequency is higher than 1 MHz, the received waveform transforms into an AC-signal on a DC offset via the rectifier to stimulate the targeted nerve tissue with internal resistance (R_{Load}). This system exhibits frequency dependent behavior that follows mainly from characteristics of the diode[1]. For the operating frequencies explored here, the diode capacitance its finite time response lead to approximately DC output without the need for a separate smoothing capacitor.

The size of the load resistance, associated with the internal resistance of the targeted nerve, changes the resonance frequency (i.e. input frequency) for the maximum output voltage (Fig. S1b). For example, the bioresorbable device without a load resistance (i.e. open circuit structure) yields the maximum output voltage ($V_{oc,max}$) of 17.4 V at an input frequency of 14.8 MHz (transmitting voltage = 3.5 V_{pp}; coil-to-coil distance (z-height) = 1.5 mm) (Fig. 1e and f), while the device with a load resistance of 5 kΩ produces the maximum output voltage of 3.0 V at an input frequency of 12.7 MHz (transmitting voltage = 3.5 V_{pp}; z-height = 1.5 mm) (Fig. S1c). The design parameters of both the Tx and Rx coils can affect the resonance frequency of the system. In this context, the device is designed to offer maximum power transfer efficiency at the resistance of sciatic nerve (~5 kΩ) (Fig. S1d and e). At a load resistance of 5 kΩ, the device shows a maximum power and efficiency of 43 mW and ~0.8 % (transmitting voltage = 16.6 V_{pp}; transmitting frequency = 12.7 MHz; z-height = 1.5 mm), respectively.

The vertical (i.e. z-height) and horizontal (i.e. misalignment) distance between the Tx and Rx coils are critical for the inductive coupling-based wireless energy transfer. Fig. S1f shows the changes in open circuit voltage (V_{oc}) as a function of vertical distance. The influence of misalignment is represented in Fig. S1g. Under small misalignment (< 3 mm), the V_{oc} (coil diameters = 12 mm; z-height = 1.5 mm) remains largely unchanged, but decreases significantly for misalignments larger than 4 mm. Increasing the z-height from 1.5 to 2.5 mm further reduces the overall V_{oc} at a given degree of misalignment. Significant reductions occur for misalignments greater than 3 mm. For the in vivo rat model, and for other practical use cases, good alignment can be easily achieved by marking the position of the receiver coil during the implantation process and adjusting the transmitting coil appropriately.

Supplementary Note 2. Limitation of existing bioresorbable encapsulation materials

b-DCPU is attractive due to its bioresorbable properties, adhesion characteristics, elastic mechanical response, negligible swelling behavior, and biocompatibility. Robust adhesion of the bioresorbable encapsulation material with itself and with other bioresorbable materials is crucial not only for ensuring mechanical integrity but also for preventing interfacial penetration of biofluids[2]. According to our previous studies of bioresorbable electronic systems[2,3], a mechanical and electrical failure can originate not only from out-of-plane water permeation through the encapsulation materials but also from in-plane water penetration through weak interfacial bonds or gaps between material layers, leading to short functional lifetimes (< 6 days). As a result, increasing the thicknesses of bioresorbable encapsulants formed with poly(lactic-co-glycolic acid) (PLGA) or Candelilla wax[3,4] does not extend the lifespan of the device significantly since such approach cannot prevent the in-plane water penetration.

Mechanical properties and swelling behaviors in biofluids are also important aspects of bioresorbable encapsulation materials. Most bioresorbable inorganic encapsulation materials, such as silicon dioxide[5,6], silicon nitride[6] and various metal oxides[7], are of limited practical utility, due to mechanical rigidity and resulting fragility[8]. Bioresorbable organic alternatives, such as silk fibroin[9,10], poly(L-lactide)[11], polylactic acid (PLA), PLGA[3], etc.[12], often exhibit swelling from water uptake and/or weak interfacial adhesion. The former can lead to failure due to fracture of the underlying device components, while the latter results in delamination[2,3]. Although engineering strategies to impart stretchability to rigid inorganic-based semiconductors[13] can be considered, in-plane water penetration at weak interfaces can limit the lifetimes.

Supplementary Note 3. Characterization of b-DCPU

The cross-linking ratio of hard segments in this polymer system provides a means to tailor the properties of b-DCPU to meet application requirements. Synthesis of b-DCPU with four different cross-linking densities (i.e. ratio of isocyanate-hydroxyl = 70, 80, 90 and 100%), named b-DCPU 80, b-DCPU 90, and b-DCPU 100, illustrate the effects. Fig. S4a shows the results of Fourier-transform infrared spectroscopy (FTIR; Nicolet 5700) measurements on uncured b-DCPU 80 (transmission mode) and cured b-DCPU 80, 90 and 100 (reflection mode). Uncured b-DCPU 80 shows a peak at 2300 cm^{-1} associated with isocyanate groups (-NCO). No isocyanate groups are present in cured b-DCPU 80, 90 and 100, consistent with reactions that consume these groups. As shown in Fig. S4b, the results of differential scanning calorimetry (DSC; Q200; a heating rate of 10°C min^{-1}) on cured b-DCPU 80, 90 and 100 reveal an absence of phase transitions between 0 to 80°C and no melting below 0°C, consistent with an amorphous character across the range of temperatures of biomedical interest (25 to 45°C). The glass transition temperature of b-DCPU increases with crosslinking density. Fig. S4c demonstrates thermal stability across the relevant temperature range through thermogravimetric analysis (TGA; a heating rate of 10°C min^{-1}). The temperature at which decomposition begins is ~300 °C, and fast weight loss occurs at ~400°C for all samples. Fig. S4d presents the transmittance spectra for wavelengths between 200 and 800 nm for b-DCPU 80 (black), 90 (red) and 100 (blue), respectively, measured by a UV/Vis/NIR spectrophotometer (LAMBDA 1050, Perkin Elmer). The results show that transmittance for wavelengths larger than 400 nm exceeds > 80% through a sample with a thickness of 200 μm. b-DCPU films exhibit hydrophobicity character, with a water contact angle of ~100°, independent of cross-linking ratio (Fig. S4e).

Supplementary Note 4. Adhesion mechanisms with b-DCPU

As a mechanism for interfacial bonding between polymers in bioresorbable electronics, permanently adjusted chemical cross-linking of thermoset materials with dynamic covalent network structure (i.e. dynamic covalent polymers) are preferred, in terms of mechanical and chemical stability[14,15], to temporary reversible bonding, including hydrogen bonds, of thermoplastic or conventional self-healing materials[16]. Dynamic covalent polymers are the recently emerged polymers that able to change their bond arrangement via reversible reaction which results in covalent interfacial bonding between different parts[14,15,17,18]. The strong adhesion between two layers (i.e. interfacial self-bonding) of bioresorbable dynamic covalent polyurethane (b-DCPU) follows from thermally activated dynamic bond exchange reactions (Fig. S5a). First, applied pressure generates conformal contact between two layers of b-DCPU. Then, at an appropriate setting temperature (~200°C), $Sn(Oct)_2$ promotes dynamic bond exchange reactions (transesterification and transcarbamoylation) and formation of new chemical bonds (ester and urethane bonds) among interfaced chains across the interfacial area (Fig. S6). The ratio of isocyanate to hydroxyl groups plays a vital role in the resulting adhesion (i.e. interfacial toughness) because the amounts of free hydroxyl groups and the relative concentration of ester moieties throughout the network matrix influence the transesterification bonding process.

Supplementary Note 5. Permeation behavior of b-DCPU

Studies of the time-dependent changes in the resistance of Mo traces (~15 µm thick) without encapsulation, encapsulated with b-DCPU 80 (~100 µm thick), and encapsulated with PLGA (65:35 (lactide:glycolide); M_w 40-75 K; ~100 µm thick), after immersion in PBS (pH 7.4; 37°C) (Fig. 2e), confirm the importance of strong interfacial adhesion on the operational stability of bioresorbable devices in biofluids. The results show that b-DCPU encapsulated Mo yields negligible changes in electrical properties over 90 days. In contrast, the resistances of Mo, without encapsulation and with PLGA encapsulation, increase with a rate of 0.05 Ω/day (up to 30 days) due to its hydrolytic degradation[7,19], and loses its conductivity after 80 and 60 days, respectively.

Gradual hydrolysis of serpentine thin-film traces of Mg (length 21.35 mm, width 150 µm, thickness ~300 nm) can be used to define the relative out-of-plane water permeability of encapsulation layers cast on top. Fig. S6 shows the time-dependent changes in the resistance of Mg traces encapsulated with 300 µm thick layers of PLGA (65:35) and b-DCPU 80, 90 and 100, after immersion in PBS (pH 7.4; 37°C). PLGA and b-DCPU 80 show similar levels of out-of-plane water permeability. Water passes through the PLGA and b-DCPU 80 and dissolves the Mg within 2-4 and 4-5 h, respectively. The water permeability of b-DCPU decreases with increasing cross-linking ratio. Here, a 1D analytical model, detailed in the Experimental Section, captures the effects of reactive diffusion of PBS with the PLGA and b-DCPU encapsulation. The diffusivity (D_{polymer}) of PLGA and b-DCPU estimated in this manner are 4.2×10^{-12} m^2 s^{-1} and 1.0×10^{-13} m^2 s^{-1} which is close to the reported diffusivity for typical biodegradable polymers[4,20].

Considering the similar level of out-of-plane water permeability between PLGA and b-DCPU 80 (Fig. S6), the resistance changes of fully encapsulated Mo (Fig. 2e) imply that the loss of conductivity in Mo with PLGA encapsulation originates not from out-of-plane water permeation but from in-plane water penetration at the interface. Much higher rate of resistance increase in PLGA encapsulated Mo than that of Mo without encapsulation after 30 days of immersion can be explained by accelerated degradation of Mo in the low pH environment created by-products associated with hydrolytic degradation of PLGA[19]. These results suggest the possibility of long-lived operation of a bioresorbable electronic devices with b-DCPU due to its strong interfacial adhesion. It must be noted that we only measure the resistance changes of polymer encapsulated Mo electrode to demonstrate the device stability in PBS (Fig. 2e), and the interconnection would not be considered in this experiment. Since biofluids affect extension and nerve contact electrodes dominantly *in vivo,* and the harvester unit, including electrical connection points, are placed in a subcutaneous pocket where much lesser biofluids exists. *In vivo* testing in rodent model shows the stability of the whole device, including b-DCPU encapsulated electrodes and interconnections.

Supplementary Note 6. Mechanical stretchability of b-DCPU

Fig. 2f shows stress-strain tensile measurements on b-DCPU 70 (black), 80 (red), 90 (orange), and 100 (olive). The corresponding Young's moduli are 0.51, 0.93, 3.12 and 3.76 ± 0.24 MPa, respectively. b-DCPU 70 and 80 exhibit elongation (i.e. strain at fracture point) of up to 340 and 230% respectively, while that of b-DCPU 90 and 100 are both larger than 170%. The results reveal an increase in elongation and a reduction in Young's modulus with a decrease in crosslink density.

Additional tests define the mechanical and electrical properties of the integrated system. Sandwiching a thin layer of a serpentine shaped Mo electrode by two layers of b-DCPU yields a mechanically robust, stretchable interconnect (Fig. S8). As demonstrated in Fig. 2g, the resistances of the structure show stable values for stretching at up to 50% strain, just prior to mechanical fracture. Finite-element analysis (FEA) results illustrate relationships between geometrical designs of the serpentine structure and the limits of elastic deformation, and show that the maximum strain in Mo is significantly less than its yield strain (0.6%) under stretching of 50% (Fig. 1g inset and Fig. S9). Fig. S10 shows the electrical stability of a b-DCPU encapsulated Mo electrode under cyclic loading conditions. Tensile fatigue tests of b-DCPU samples show the almost consistent behaviour on stretching and releasing, with negligible hysteresis in the strain range from 0-20% during 2000 cycles (Fig. S10a). The resistances of b-DCPU encapsulated Mo electrodes in stretched and compressed states show stable values for 10,000 cycles (with a rate of 5 mm/s) to tensile strains of +20% (stretching) and -20% (compression involving buckling), similar to those expected in the muscle movement of a rat leg[21,22] (Fig. S10b).

Supplementary Note 7. Degradation behavior of b-DCPU

The soft segment, PCL, and hard segment, aliphatic diisocyanate, in the b-DCPU are biodegradable[23,24]. The degradation of b-DCPU involves three stages: (1) water absorption; (2) chain scission by the hydrolysis of ester and urethane groups; and (3) dissolution of water-soluble entities[25]. During immersion in PBS (pH 7.4; 37°C), b-DCPU exhibits negligible swelling with water uptake of 2 ± 0.1% after one day of immersion. By comparison, PLGA 65:35 absorbs more than 8 wt% of water after 4 h of immersion (Fig. S11). Fig. S12a shows the storage modulus of 25-mm-diameter b-DCPU 80 disks (thickness: ~200 μm) immersed in PBS (pH 7.4) at 85°C for 0, 2, 5, 10, 20 and 30 days, respectively. The storage modulus decreases with time of immersion (Fig. S12), consistent with gradual delinking of the polymer network and chemical degradation. Fig. S13 shows the degradation behavior of b-DCPU in PBS (pH 7.4) at temperatures of 23, 37, 60 and 80°C. The b-DCPU film degrades gradually in PBS at 37°C over a year and to completion in 80 days at 80°C. The b-DCPU 80 degrades with a rate of ~4.78×10^{-5} g/day (for the first 24 days) in PBS (pH 7.4) at physiological temperatures (37°C). The dissolution rate of b-DCPU 80 for the first 7 days at 80°C (~1.28×10^{-4} g/day) is approximately 4 times higher than that at 23°C (~3.34×10^{-5} g/day). This timescale can also be controlled by manipulating the surface area to volume ratio.

The rate of degradation depends on the chemistry of its soft segment structure composed of the polyols. Hydrophilic PEG, as another possible soft segment, typically degrades more quickly than hydrophobic PCL under physiological conditions[26]. As a result, the degradation rate of the b-DCPU can be controlled by adding hydrophilic PEG and tuning its composition. Compared to the degradation behavior of b-DCPU 80, PEG containing b-DCPU with weight ratio (PCL:PEG) of 22-58 degrades much faster, showing complete degradation within 11 days in the same physiological condition (Fig. 2h).

Supplementary Note 8. Biocompatibility and toxicity of b-DCPU

The non-toxicity of PCL-triol, HDI, and resulting polyurethane network is well-established[27]. Although tin-based catalysts are FDA-approved, studies of long-term liberation and physiological effects of tin-ions from large tissue-embedded scaffold-matrices have not been previously performed. The following describes comprehensive biocompatibility and toxicity tests on $Sn(Oct)_2$ containing b-DCPU.

(1) *In vitro* biocompatibility

Studies of *in vitro* biocompatibility of b-DCPU throughout the degradation process involve three different b-DCPU samples: (1) normal b-DCPU 80 (Pristine); (2) degraded b-DCPU 80 with gel-type texture (Degradation or Deg.) degraded in PBS (pH 7.4) at 95°C for a month; (3) fully dissolved b-DCPU 80 solution (Solution or Sol.). Live/dead staining of cultured mice fibroblasts in different conditions for 3 days demonstrate no significant influence on *in vitro* cell survival on Pristine, Deg. and Sol., as compared to tissue culture polystyrene (TCPS), indicating that b-DCPU and its degradation by-products do not have any influence on cell survival (Fig. 2i). Normalized *in vitro* viability assay data present no signs of cytotoxicity for all the groups with b-DCPU (Fig. 2j).

(2) *In vivo* biocompatibility

Histological analysis shows that, after 4 weeks of implantation, the b-DCPU generates inflammatory reactions similar to those produced by high-density polyethylene (HDPE, FDA-approved nontoxic control) (Fig. S19). Hematoxylin and Eosin (H&E) stained sections of implant sites surrounding tissues show comparable levels of immune cell infiltration between b-DCPU and HDPE-implanted tissues at day 28. Masson Trichrome staining revealed identical tissue fibrosis around implanted material. Immunohistochemical staining of skin four weeks after implantation indicates identical monocyte (CD68) and leukocyte (CD45) inflammatory cell infiltration at the material-tissue interface. The results in Fig. S20a show that the changes in weight between mice with PLGA implants, as controls, and those with b-DCPU implants are similar throughout the 4-weeks period of observation. Following implantation, the mice behave normally with no substantial skin necrosis or swelling for up to 4 weeks. The result indicates minimal differences, consistent with the absence of toxic effects. The results of complete blood chemistry tests provide a comprehensive understanding of the health of the mice (Fig. S20b and c). Blood levels of enzymes and electrolytes, which serve as indicators of organ-specific diseases, fall within the confidence intervals of control values. For example, normal levels of alanine aminotransferase, cholesterol and triglyceride, phosphorus and urea nitrogen, calcium, albumin and total proteins indicate the absence of disorders in the liver, heart, kidney, bone and nerve, and good overall health, respectively.

Supplementary Note 9. Conventional neuromuscular electrical stimulation systems

Programs of neuromuscular electrical stimulation (NMES) appear to be acceptable to patients and have led to improvements in muscle strength, increasing range of motion, reducing edema, decreasing atrophy, healing tissue, and decreasing pain[28]. The effects of NMES therapy are expected on neuromuscular function in 3 populations: (1) preservation of neuromuscular function in patients during disuse; (2) partial restoration of neuromuscular function in patients after disuse; and (3) improvement of neuromuscular function in able-bodied individuals with "normal" function[29]. For example, applying NMES directly over a nerve trunk increased motor unit compared with a muscle belly[30]. In addition, as a strategic therapy for neuromuscular disorders, direct NMES significantly increased the number and size of AChRs clusters available for neuromuscular junction formation[31]. It is also reported that denervated muscles that received daily electrical muscle stimulation for 3 months had significantly greater numbers of reinnervated motor units, and improved behavioral outcomes[32]. In large animals, daily NMES for 8 weeks reversed the muscle fiber atrophy produced by complete denervation while avoiding a shift to a slow (type 1) fiber type[33].

NMES occurs through depolarization of the motor nerves (rather than the muscle fibers directly) via transcutaneous, percutaneous, and implantable electrodes. As a non-invasive method, transcutaneous electrical stimulation devices apply current to muscles through electrodes on the skin. Due to the high impedance of the skin, transcutaneous muscle stimulation requires voltages with magnitudes ten times larger than those of implantable electrodes[34]. Hence, transcutaneous devices require much higher levels of power to induce muscle contractions for cases of traumatic nerve injuries. Implantable devices provide a direct means for neuromuscular stimulation via nerve cuff electrodes. A major disadvantage is that the associated surgical retrieval procedures subject patients to distress and expose them to the potential for additional complication[35,36]. In addition, conventional NMES mainly focuses on innervated muscle rehabilitation. For cases of damage to lower motor neurons or neuromuscular junctions (i.e. partial or complete denervation), the therapeutic benefit of NMES to the denervated muscle via implantable electrodes remains unclear[34].

Supplementary Note 10. Modeling of reactive diffusion for polymer encapsulated Mg

The reactive-diffusion model (Fig. S23) can be expressed as

$$\begin{cases} D_{Mg}\frac{\partial^2 w}{\partial y^2} - k_{Mg}w = \frac{\partial w}{\partial t}, 0 \le y \le h_0 \\ D_{polymer}\frac{\partial^2 w}{\partial y^2} = \frac{\partial w}{\partial t}, h_0 \le y \le h_0 + h_{polymer} \end{cases} \quad (1)$$

where the water concentration w is solved as a function of time t and position y. The diffusivity (D_{Mg}) and reaction constant (k_{Mg}) of water in Mg are considered for $0 \le y \le h_0$. Due to the slow reaction process between the polymer encapsulation and water, only the diffusivity of water in polymer ($D_{polymer}$) is considered for $h_0 \le y \le h_0 + h_{polymer}$, thereby reducing the equation to standard diffusion.

When the specimen is submerged in the aqueous solution, the water concentration at the interface between the top surface of the polymer encapsulation and water is constant, i.e.,

$$w|_{y=h_0+h_{polymer}} = w_0 \quad (2)$$

the water flux at the bottom surface of Mg is zero

$$\left.\frac{\partial w}{\partial y}\right|_{y=0} = 0 \quad (3)$$

and the water concentration w at time $t = 0$ is zero.

$$w|_{t=0} = 0 \quad (4)$$

At the polymer/Mg interface, the concentration and flux of water molecules follows a continuity condition such that

$$w|_{y=h_0-0} = w|_{y=h_0+0} \quad (5)$$

$$D_{Mg}\left.\frac{\partial w}{\partial y}\right|_{y=h_0-0} = D_{polymer}\left.\frac{\partial w}{\partial y}\right|_{y=h_0+0} \quad (6)$$

The solution of the reactive-diffusion problem is obtained by following the method presented elsewhere[4,37]. The water concentration (w) as a function of time (t) and position (y) is obtained as

$$w(t, y) = w_0\left[\sum_1^\infty C_n e^{-\lambda_n t} f_n(y) + g(y)\right] \quad (7)$$

Where the eigenvalues (λ_n) are determined from

$$\cos\left(\sqrt{\frac{\lambda - k_{Mg}}{D_{Mg}}}h_0\right)\cos\left(\sqrt{\frac{\lambda}{D_{polymer}}}h_{polymer}\right) = \sqrt{\frac{D_{Mg}(\lambda - k_{Mg})}{D_{polymer}\lambda}}\sin\left(\sqrt{\frac{\lambda - k_{Mg}}{D_{Mg}}}h_0\right)\sin\left(\sqrt{\frac{\lambda}{D_{polymer}}}h_{polymer}\right) \tag{8}$$

The functions $f_n(y)$ and $g(y)$ are given as

$$f_n(y) = \begin{cases} \sin\left(\sqrt{\frac{\lambda_n}{D_{polymer}}}h_{polymer}\right)\cos\left(\sqrt{\frac{\lambda_n - k_{Mg}}{D_{Mg}}}y\right), & 0 \le y \le h_0 \\ \cos\left(\sqrt{\frac{\lambda_n - k_{Mg}}{D_{Mg}}}h_0\right)\sin\left[\sqrt{\frac{\lambda_n}{D_{polymer}}}\left(h_0 + h_{polymer} - y\right)\right], & h_0 \le y \le h_0 + h_{polymer} \end{cases} \tag{9}$$

$$g(y) = \begin{cases} \frac{1}{\sqrt{\frac{D_{Mg}k_{Mg}h_{polymer}^2}{D_{polymer}^2}}\sinh\sqrt{\frac{k_{Mg}h_0^2}{D_{Mg}}} + \cosh\sqrt{\frac{k_{Mg}h_0^2}{D_{Mg}}}}\cosh\left(\sqrt{\frac{k_{Mg}}{D_{Mg}}}y\right), & 0 \le y \le h_0 \\ 1 - \frac{1}{1 + \sqrt{\frac{D_{polymer}^2}{D_{Mg}k_{Mg}h_{polymer}^2}}\coth\left(\sqrt{\frac{k_{Mg}h_0^2}{D_{Mg}}}\right)}\frac{h_0 + h_{polymer} - y}{h_{polymer}}, & h_0 \le y \le h_0 + h_{polymer} \end{cases} \tag{10}$$

Using the initial condition, the coefficient (C_n) can be determined as

$$C_n = \frac{-\frac{2}{\lambda_n}\sqrt{\lambda_n D_{polymer}}\cos\left(\sqrt{\frac{\lambda_n - k_{Mg}}{D_{Mg}}}h_0\right)}{h_0\sin^2\left(\sqrt{\frac{\lambda_n}{D_{polymer}}}h_{PA}\right)\left[1 + \frac{\sin\left(2\sqrt{\frac{\lambda_n - k_{Mg}}{D_{Mg}}}h_0\right)}{2\sqrt{\frac{\lambda_n - k_{Mg}}{D_{Mg}}}h_0}\right] + h_{polymer}\cos^2\left(\sqrt{\frac{\lambda_n - k_{Mg}}{D_{Mg}}}h_0\right)\left[1 - \frac{\sin\left(2\sqrt{\frac{\lambda_n}{D_{polymer}}}h_{polymer}\right)}{2\sqrt{\frac{\lambda_n}{D_{polymer}}}h_{polymer}}\right]} \tag{11}$$

The mass of dissolved Mg (per unit volume) is $\frac{k_{Mg}wM_{Mg}}{qM_{H_2O}}$, where the term $k_{Mg}w$ corresponds to the mass of water that reacts at a given location (per unit volume), M_{Mg} and M_{H_2O} are the molecular mass of Mg and water, respectively, and $q = 2$ are the two water molecules that react with each Mg atom in the process. Integrating $\frac{k_{Mg}wM_{\mathrm{Mg}}}{q\rho_{\mathrm{Mg}}M_{H_2O}}$, where ρ_{Mg} is the density of Mg, over both time *t* and thickness *y* gives the volume of Mg that decreased in the process.

The normalized Mg thickness is given as

$$\frac{h(t)}{h_0} = 1 - \frac{w_0 M_{\mathrm{Mg}}}{q \rho_{\mathrm{Mg}} M_{H_2O}} k_{Mg} \left[\frac{1}{\sqrt{\frac{D_{Mg} k_{Mg} h_{polymer}^2}{D_{polymer}^2}} \sinh \sqrt{\frac{k_{Mg} h_0^2}{D_{Mg}}} + \cosh \sqrt{\frac{k_{Mg} h_0^2}{D_{Mg}}}} t \frac{\sinh \sqrt{\frac{k_{Mg} h_0^2}{D_{Mg}}}}{\sqrt{\frac{k_{Mg} h_0^2}{D_{Mg}}}} \right. \\ \left. + \sum_{n=1} \frac{C_n}{\lambda_n} \left(1 - e^{-\lambda_n t}\right) \frac{\sin\left(\sqrt{\frac{\lambda_n - k_{Mg}}{D_{Mg}} h_0^2}\right)}{\sqrt{\frac{\lambda_n - k_{Mg}}{D_{Mg}} h_0^2}} \sin\left(\sqrt{\frac{\lambda_n}{D_{polymer}} h_{polymer}^2}\right) \right] \tag{12}$$

Supplementary Table 1. The alive response after injury at lower limb nerve.

	EMG response*	Myelin degradation**	Basal lamina***
**Mice**	24 hours[38]	2-3 days[39,40]	intact
**Rat**	36 hours[41]	7-10 days[42]	intact
**Human**	7-11 days[43,44]	N/A (probably 2-3 weeks)	N/A

*Electromyograms (EMG) response: electrophysiology discontinuation / **Myelin degradation: conductive substance discontinuation / ***Basal lamina: remained soft tissue connection

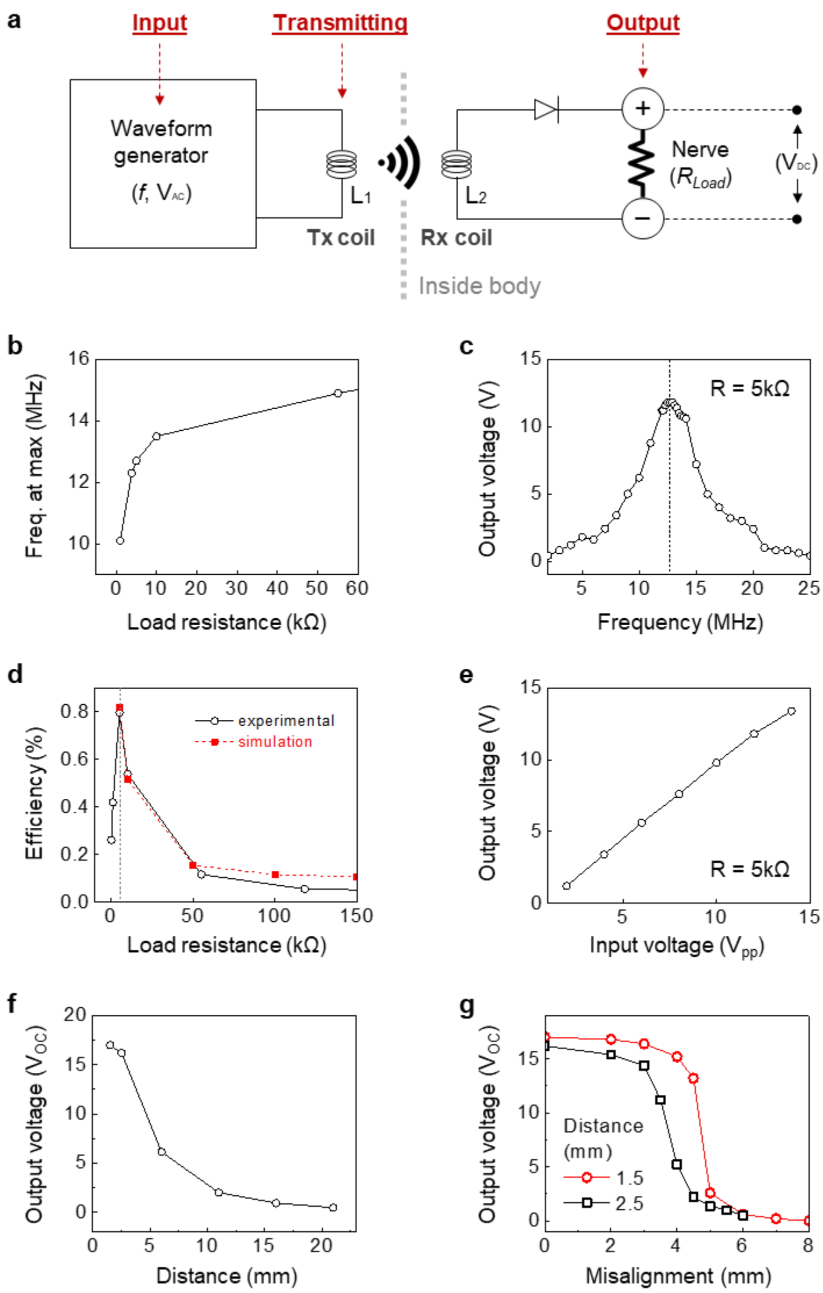

Supplementary Fig. 1 | Electrical performance characteristics of the wireless power transfer system.

a, Schematic illustration of the circuit diagram for radiofrequency (RF) wireless power transfer. (Tx: transmitting coil; Rx: receiving coil). **b,** Changes in input frequency (i.e. resonance frequency) at the maximum output voltage with load resistance. **c,** Output voltage of the bioresorbable device with load resistance of 5 kΩ as a function of transmitting frequency. At the resonance frequency (12.7 MHz) of the Rx coil (transmitting voltage = 12 V_{pp}), the device produces a maximum output voltage of 11.8 V. **d,** (black) Experimental and (red) simulation results for the energy transfer efficiency (transmitting voltage = 16.6 V_{pp}; transmitting frequency = 12.7 MHz;) as a function of load resistance at a Tx-Rx coil distance of ~1.5 mm. **e,** Output voltage (input frequency = 12.7 MHz; load resistance = 5 kΩ) as a function of input

voltage. The output voltage is determined by the waveform generator that delivers power to the antenna. **f,** Open circuit voltage (V_{oc}) as a function of the distance between the Tx and Rx coils (input voltage = 3.5 V_{pp}; input frequency = 14.8 MHz). **g,** Experimental results of V_{oc} as a function of misalignment distance between the Tx and Rx coils (input voltage = 3.5 V_{pp}; input frequency = 14.8 MHz). Vertical coil-to-coil distance (z-height) are (red, circle) 1.5 mm and (black, square) 2.5 mm.

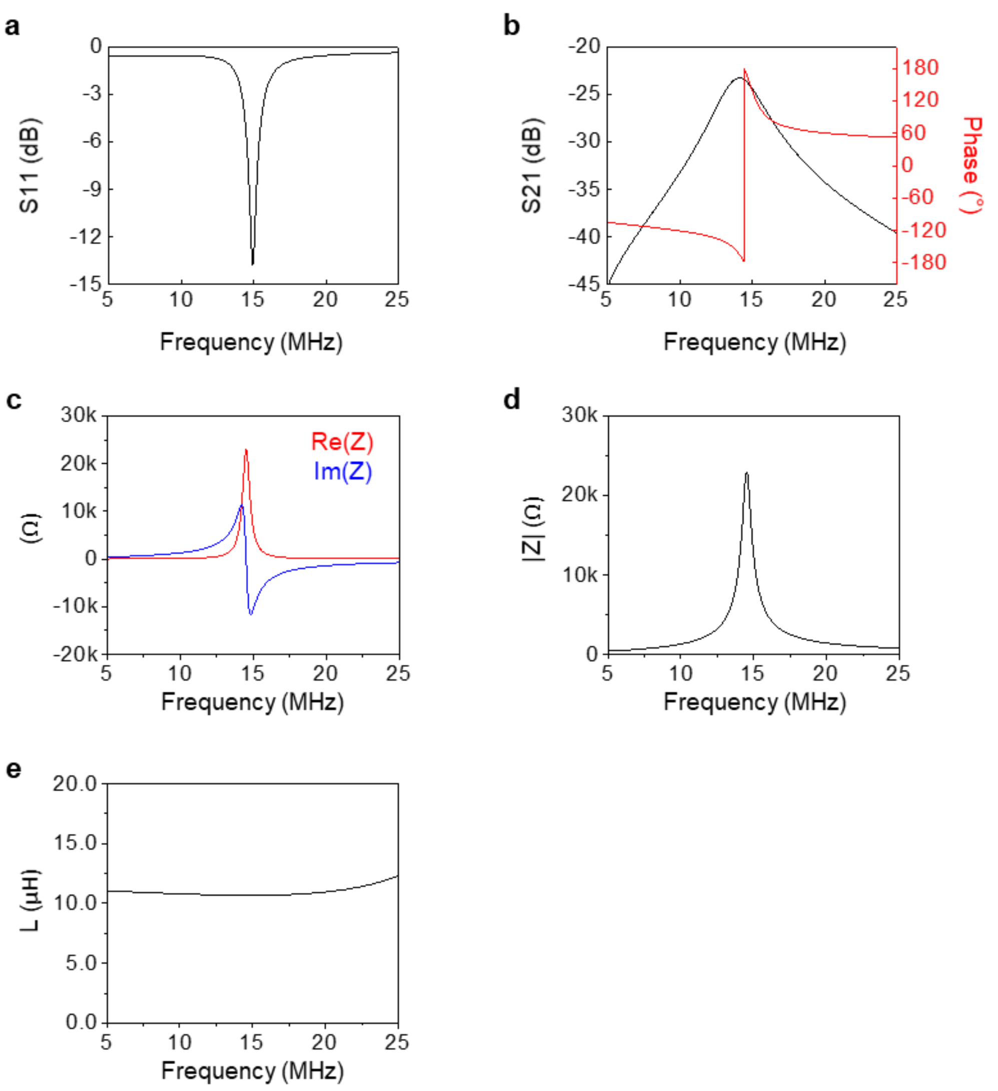

Supplementary Fig. 2 | Electrical characteristics of the transient inductor.

a, Simulated frequency-dependent S_{11} parameter of the wireless system for a separation distance of 1.5 mm. **b,** Simulated S_{21} parameter (black) and phase (red) as a function of frequency for the wireless system with a separation distance of 1.5 mm and a load resistance R = 5000 Ω. **c,** The frequency-dependent impedance (Z). (red) The real part of the impedance is the resistance, whereas (blue) the imaginary part is the reactance of the wireless stimulator. **d,** The magnitude of the impedance |Z| in c. **e,** the frequency-dependent inductance (L) of the wireless stimulator. (Re: real part of the impedance; lm: imaginary part of the impedance)

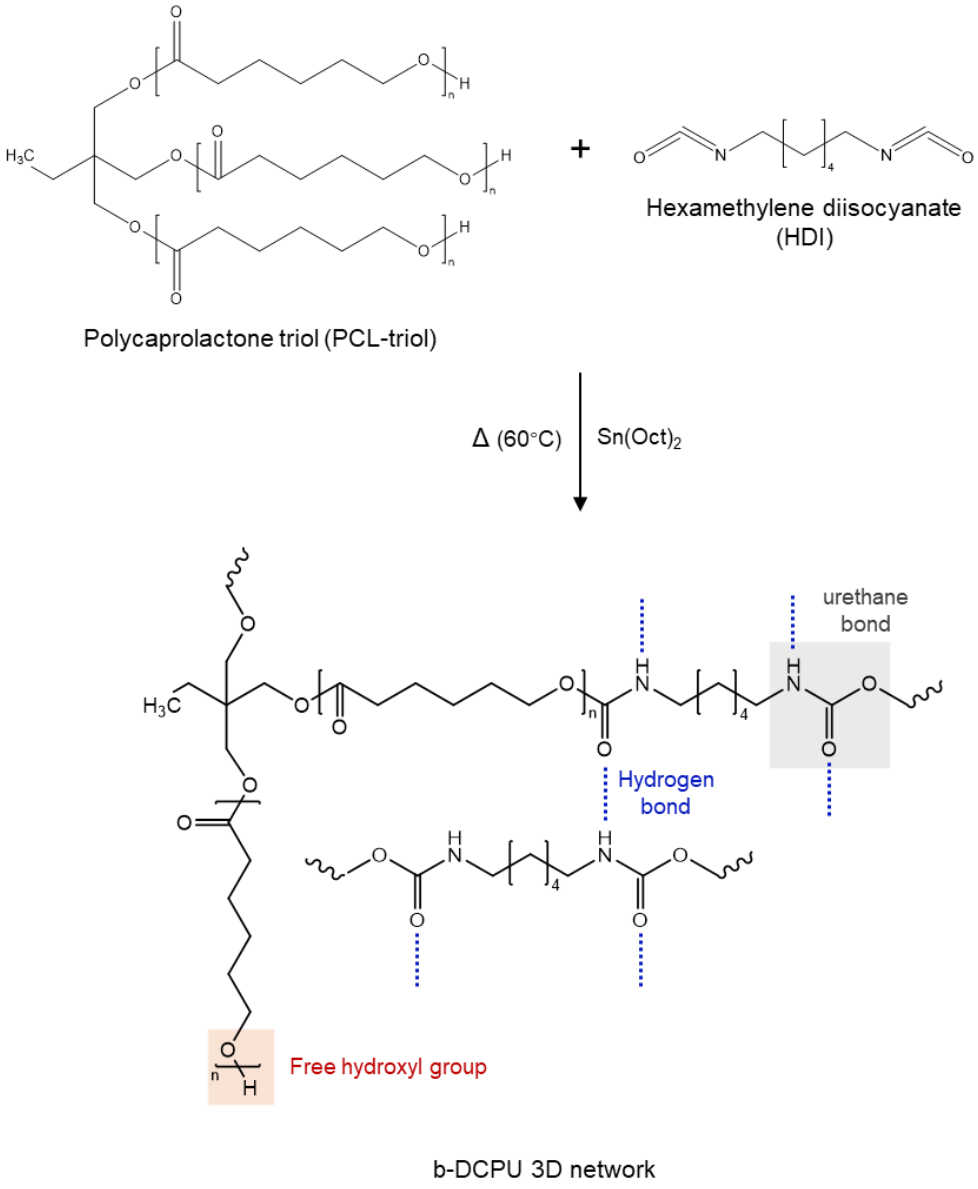

Supplementary Fig. 3 | Chemistry associated with the synthesis of b-DCPU.

Schematic illustration and molecular structures of the precursors (polycaprolactone triol, PCL-triol; hexamethylene diisocyanate, HDI) and resulting b-DCPU 3D network. The 3D network consists of chemical cross-linking through urethane bonds and physical cross-linking through hydrogen bonds and.

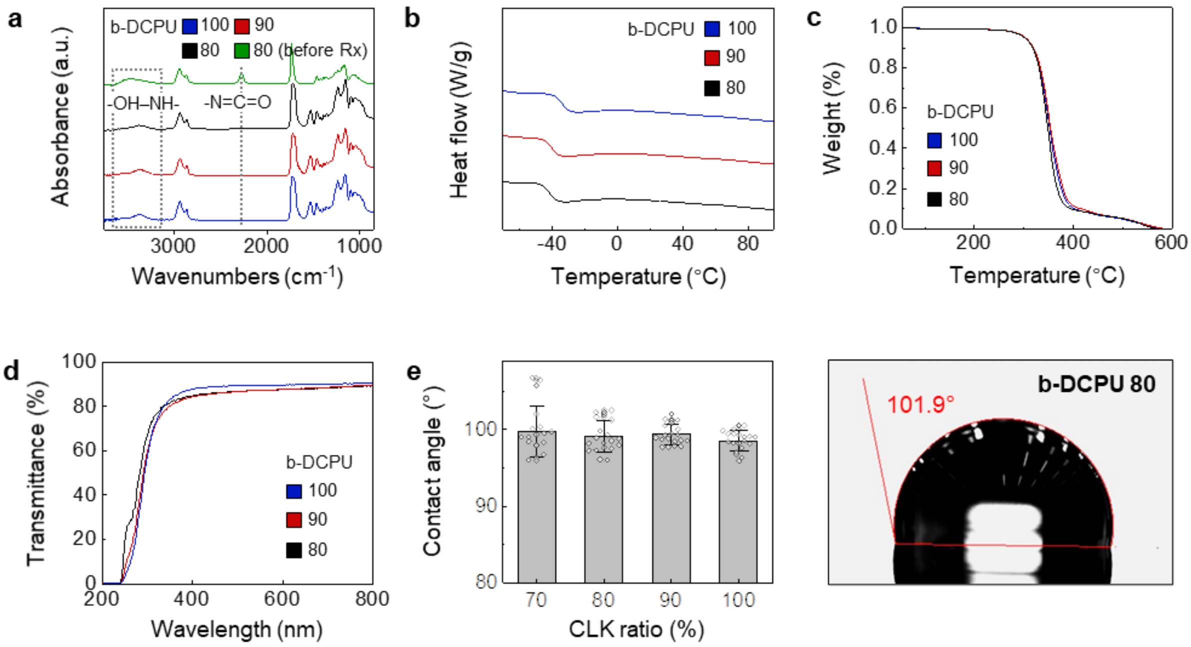

Supplementary Fig. 4 | Characteristics of b-DCPU.

a, Fourier-transform infrared spectroscopy (FT-IR). **b,** Differential scanning calorimetry (DSC). **c,** Thermogravimetric analysis (TGA). **d,** Transmittance of b-DCPU. **e,** Water contact angle (left) of b-DCPU with various cross-linking ratio and representative photograph (right) for the water contact angle of a b-DCPU 80 film. Data are presented as mean values ± s.e.m. $n > 10$ independent samples. Data available in source data file.

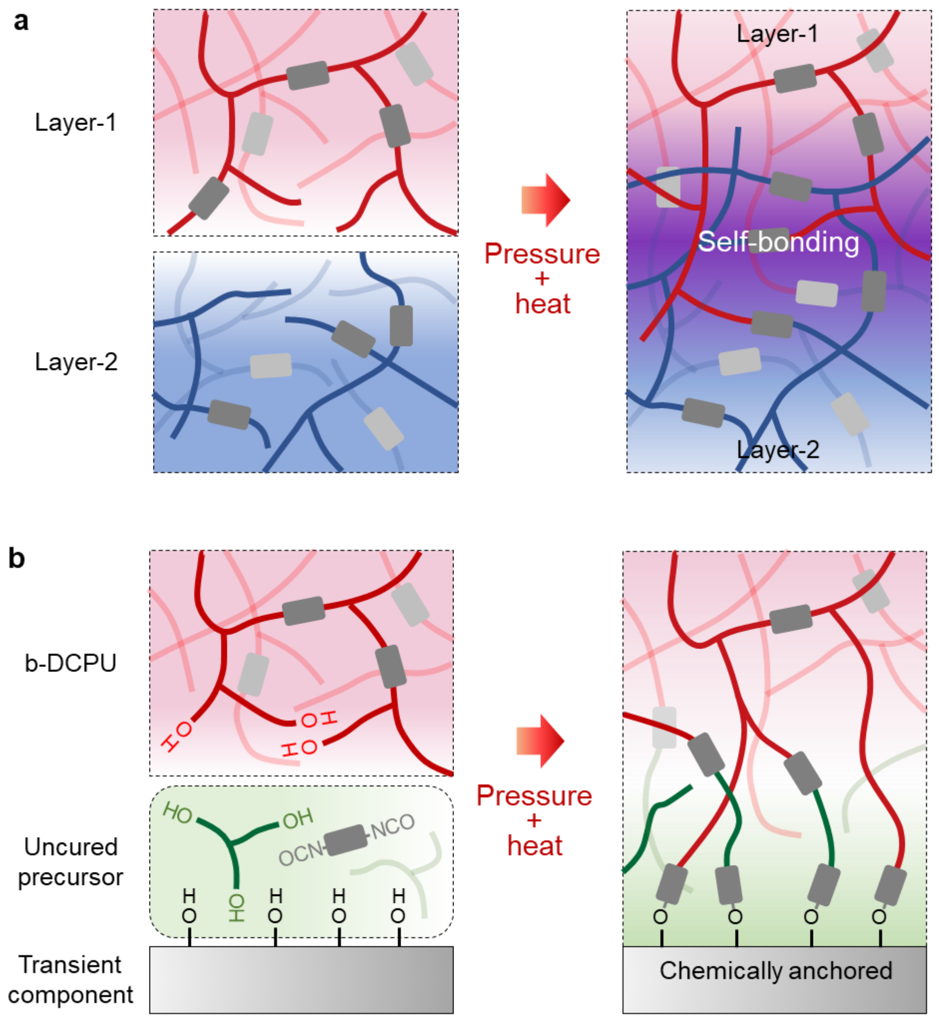

Supplementary Fig. 5 | Schematic illustrations of bonding mechanisms associated with b-DCPU.

a, Schematic illustration of the adhesion process between two layers of b-DCPU under pressure and heating: (1) pressure generates conformal contact between two layers of b-DCPU (2) heating leads to chemical bonds among chains by dynamic bond exchange reaction, resulting in 'self-bonding' between the layers. **b,** Schematic illustration of the adhesion process between b-DCPU and other bioresorbable materials (Si and metals) under heating and pressure. Pre-treatment of oxygen plasma increases hydroxyl groups (-OH) on the surfaces of bioresorbable components. Between b-DCPU and a plasma-treated component, the uncured precursor presents HDI as a cross-linking agent that enables chemical anchoring between hydroxyl groups in both the b-DCPU and the component.

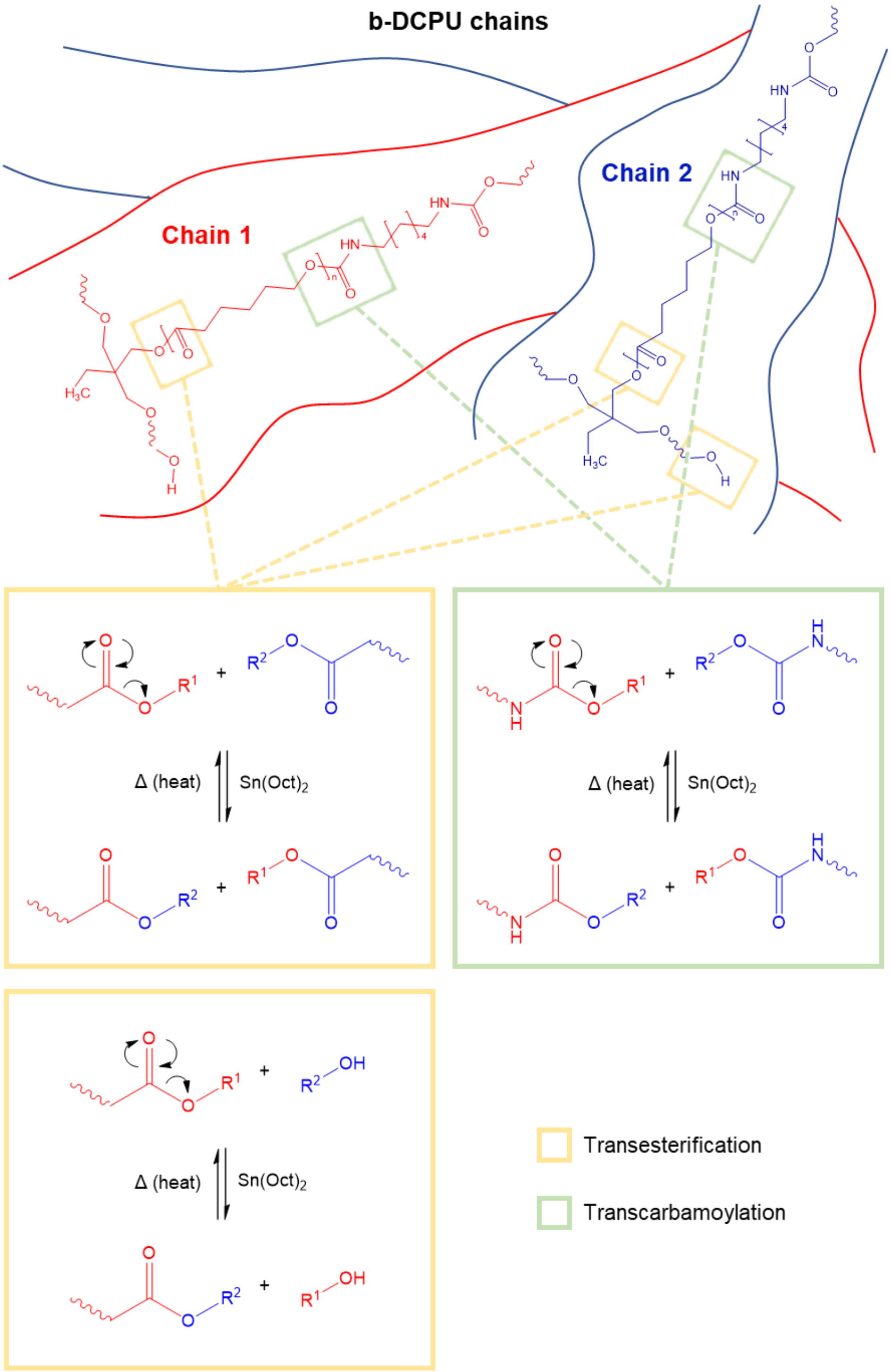

Supplementary Fig. 6 | Schematic illustration of the thermally activated catalytic bond exchange reaction.

Transesterification (left, orange box) and transcarbamoylation (right, green box) reaction among interfaced chains across the interfacial area of two b-DCPU layers.

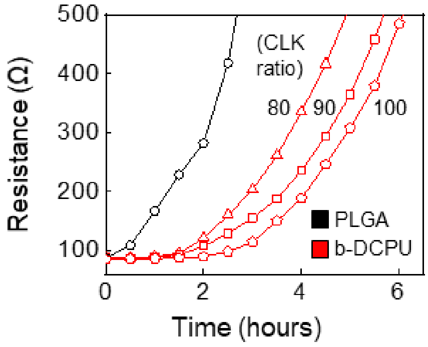

Supplementary Fig. 7 | Water permeation properties of b-DCPU and PLGA.

Permeation properties of bioresorbable PLGA 65:35 (lactide:glycolide) (black) and b-DCPU 80, 90, 100 (red) with the thickness of 300 µm as measured by the time evolution of the resistance of a thin film trace of 300 nm Mg at 37°C in PBS (pH 7.4). PBS that penetrates though the encapsulation layer dissolves the Mg trace, thereby increasing its resistance.

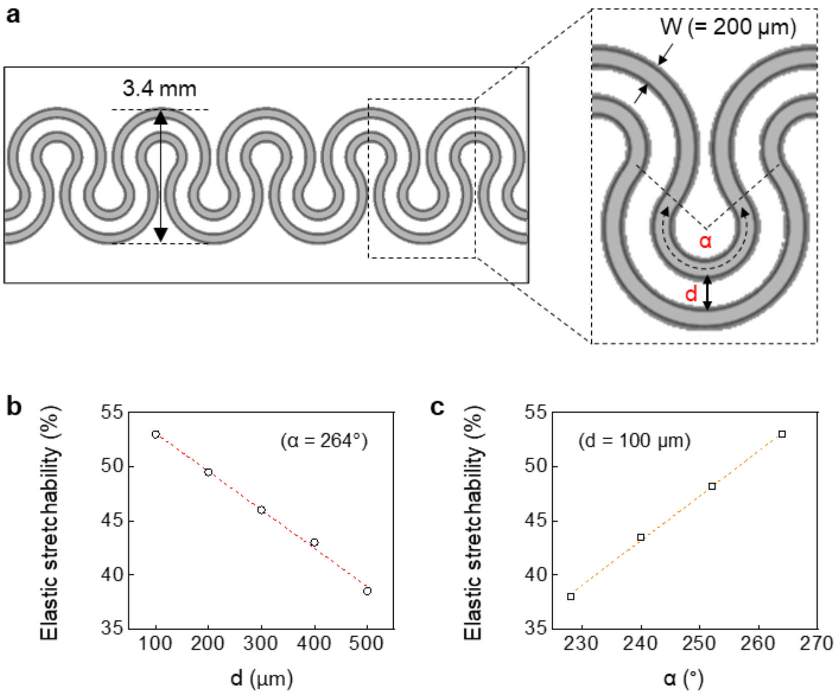

Supplementary Fig. 8 | Design optimization for serpentine-shaped electrodes based on FEA.

a, Schematic illustration of the serpentine shaped Mo electrodes. The height of the serpentine structure and width (*W*) of Mo strips are fixed to 3.4 mm and 200 μm, respectively. The Mo electrode lies near the mechanical neutral plane as it is sandwiched between 50 μm thick layers of b-DCPU. *α* and *d* indicate the center angle of the arcs and the spacing between two Mo wires, respectively. **b,** Simulated elastic stretchability of electrodes with *α* of 264° as a function of *d*. **c,** Simulated elastic stretchability of electrodes with *d* of 100 μm as a function of *α*.

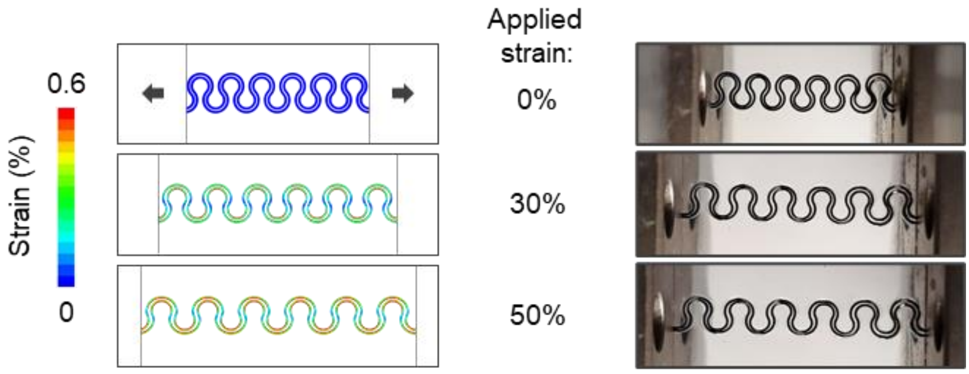

Supplementary Fig. 9 | Mechanical stretchability of b-DCPU encapsulated Mo serpentine electrodes.

Finite-element analysis (FEA) and experimental results for b-DCPU 80 encapsulated serpentine electrodes (Mo) with a uniaxial tensile strain of 0, 30 and 50% (b-DCPU/Mo/b-DCPU, 200 μm thick).

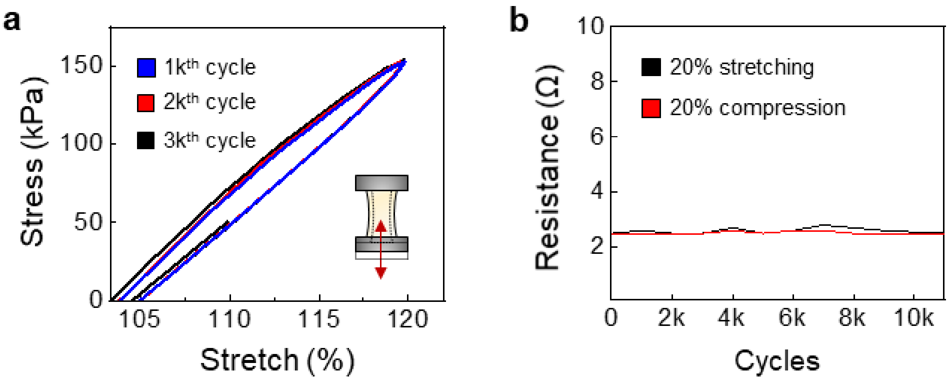

Supplementary Fig. 10 | Mechanical and optical properties of bioresorbable polyurethane.

a, Fatigue tests of b-DCPU 80. **b.** Measured changes in resistance of b-DCPU 80 encapsulated serpentine electrodes (Mo) during 10,000 cycles (5 mm/s rate) of uniaxial stretching (strain: 20%) and compression (strain: 20%).

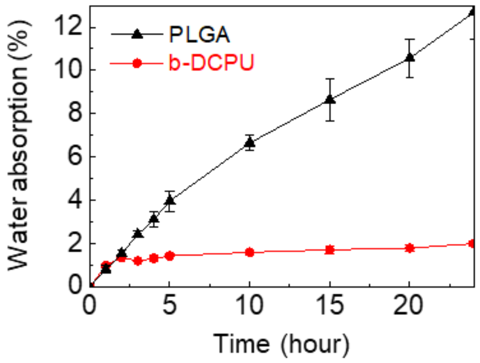

Supplementary Fig. 11 | Swelling properties of PLGA and b-DCPU.

Kinetics of water absorption of PLGA 65:35 (black) and b-DCPU 80 (red) samples after immersion in PBS (pH 7.4) at 37°C. The water uptake during the first hour was over 2% of the sample weight for both PLGA and b-DCPU. The water uptake of PLGA continues to increase with time, while b-DCPU shows a saturation behavior after 2 hours. The results are shown as means ± s.e.m. *n* = 5 independent samples.

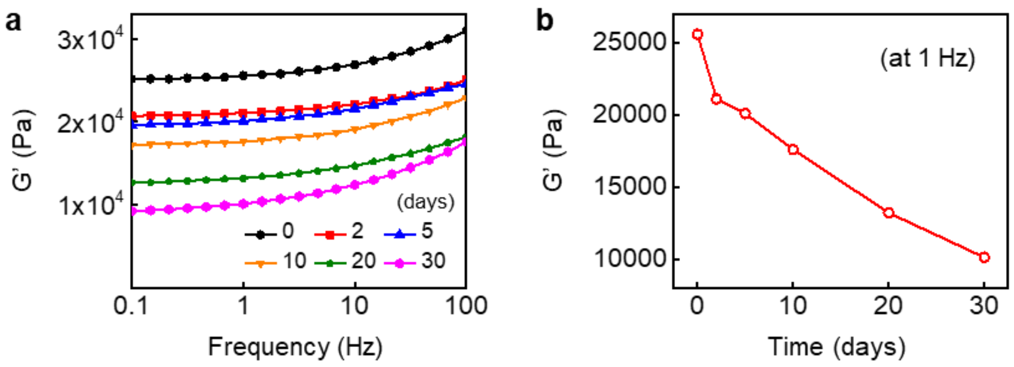

Supplementary Fig. 12 | Bioresorbable behavior of b-DCPU 80 - rheological performance and changes in mechanical properties.

a. The storage modulus (G') of b-DCPU 80 changes as a function of scanning frequency (oscillatory sweep at 5% amplitude gamma). Disc type samples were immersed in PBS (pH 7.4) at 85°C for 0, 2, 5, 10, 20 and 30 days. **b.** Changes in storage modulus at 1 Hz as a function of immersion time.

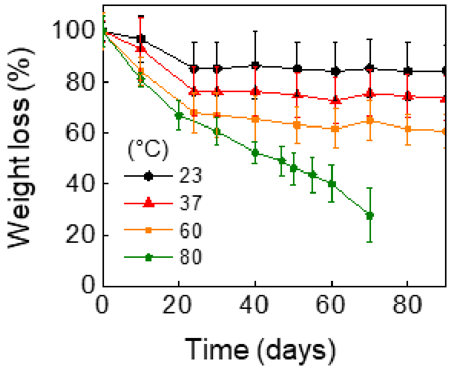

Supplementary Fig. 13 | Dissolution kinetics of bioresorbable encapsulation.

Measured changes in weight as a function of the time of immersion of b-DCPU 80 in PBS (pH 7.4) at 23 (black circle), 37 (red triangle), 60 (orange square), and 80°C (olive pentagon). The results are shown as means ± s.e.m. n = 5 independent samples per groups.

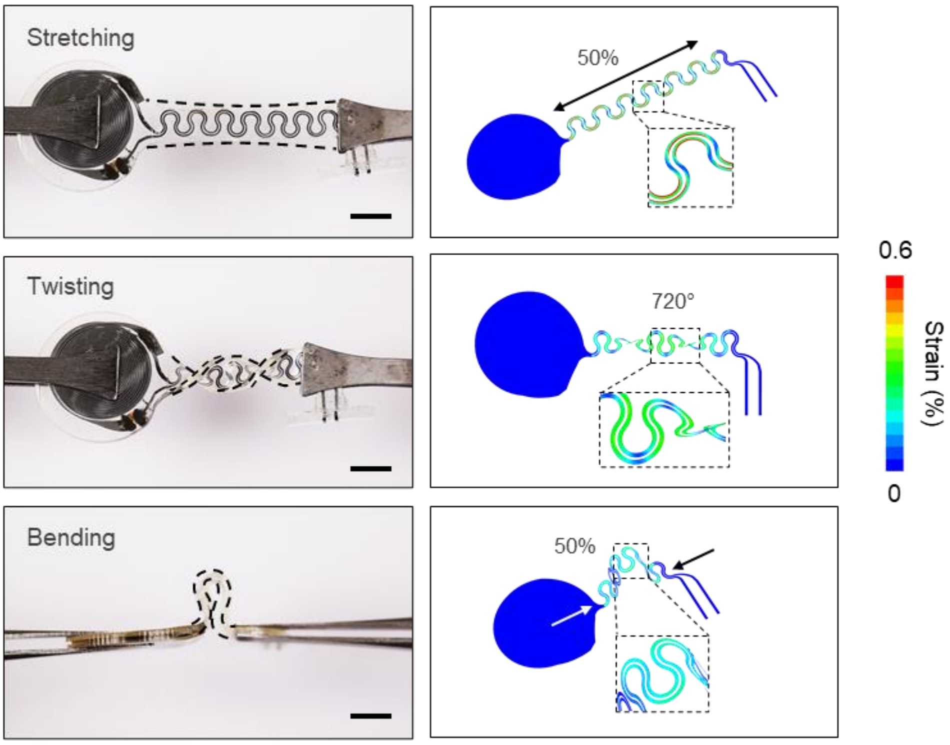

Supplementary Fig. 14 | Mechanical reliability of the bioresorbable electrical stimulator.

(Left) Photographic images of b-DCPU-encapsulated serpentine electrodes during uniaxial stretching, twisting and bending. Scale bar indicates 10 mm. (Right) Corresponding finite-element analysis (FEA) results for the integrated devices during stretching (50%), twisting (720°) and bending (50%).

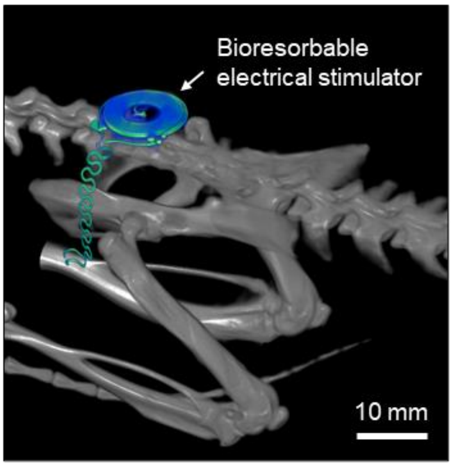

Supplementary Fig. 15 | Three-dimensional computed tomography images of a mouse collected 1 week after the implantation of a bioresorbable electrical stimulator.

The image indicates the position of the nerve cuff and receiver.

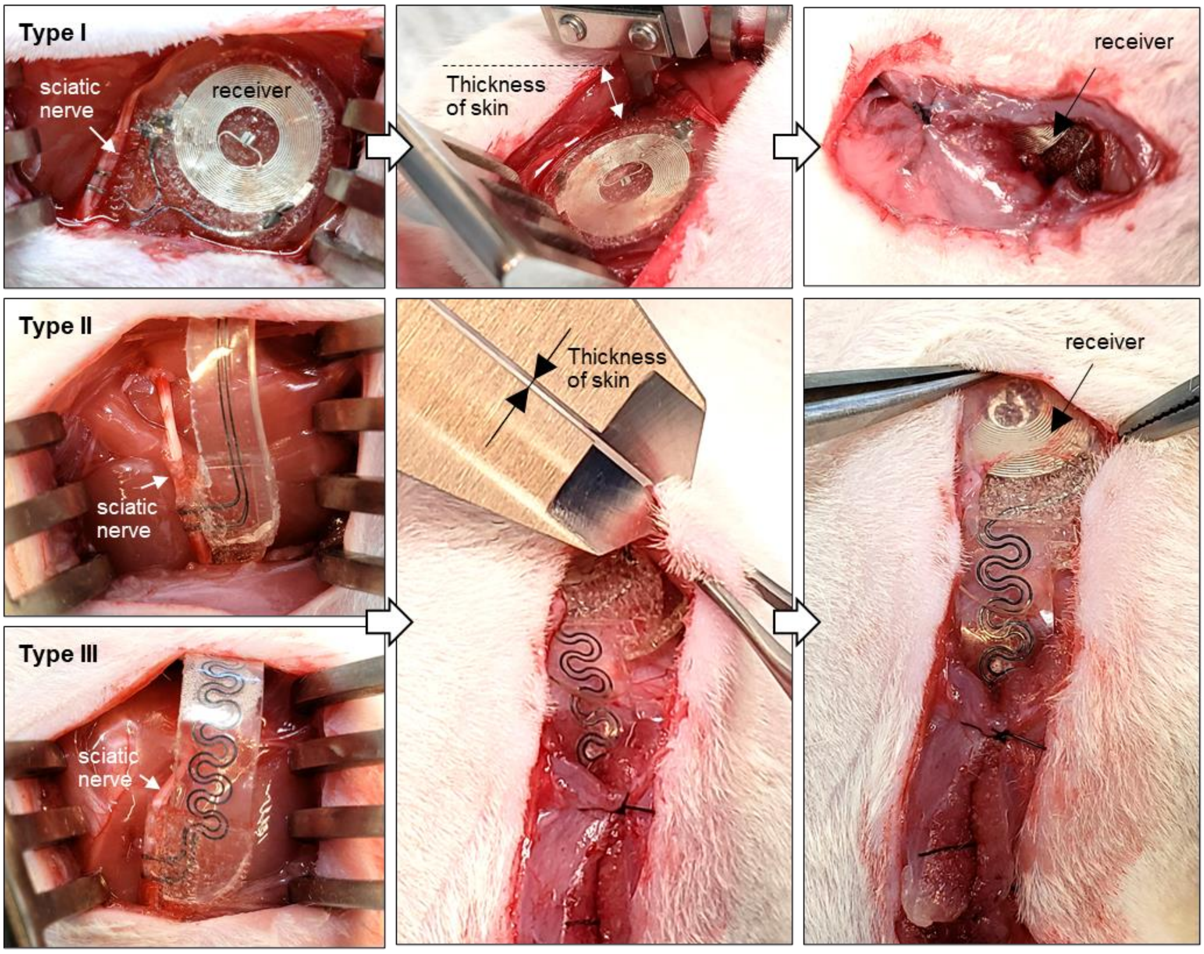

Supplementary Fig. 16 | Photographs of three different designs for the Mo electrodes.

The bioresorbable electrical stimulator without extension (Type I), with linear extension (Type II), and with the serpentine extension (Type III). A vernier caliper determines the distance between receiver and transmission coil.

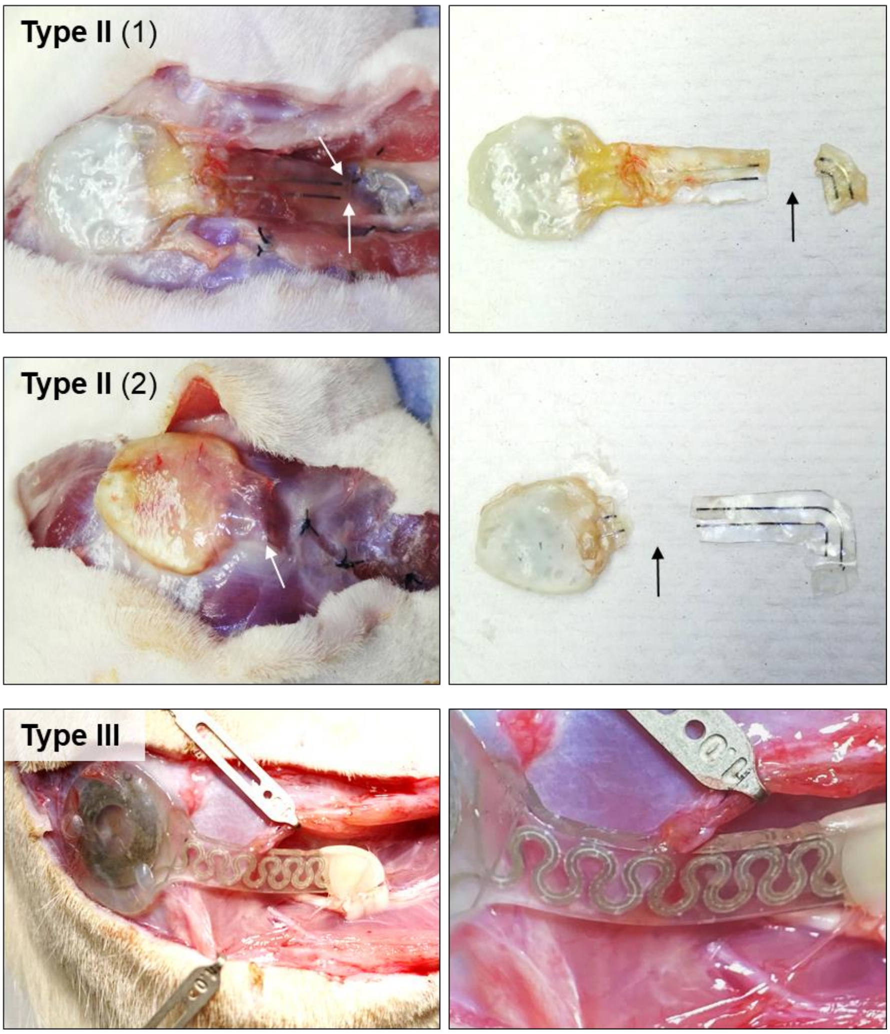

Supplementary Fig. 17 | Photographs of bioresorbable electrical stimulators with different designs for the extension electrodes.

Mechanical failure of Type II devices at receiver and nerve cuff parts, and Type III device without any mechanical failure.

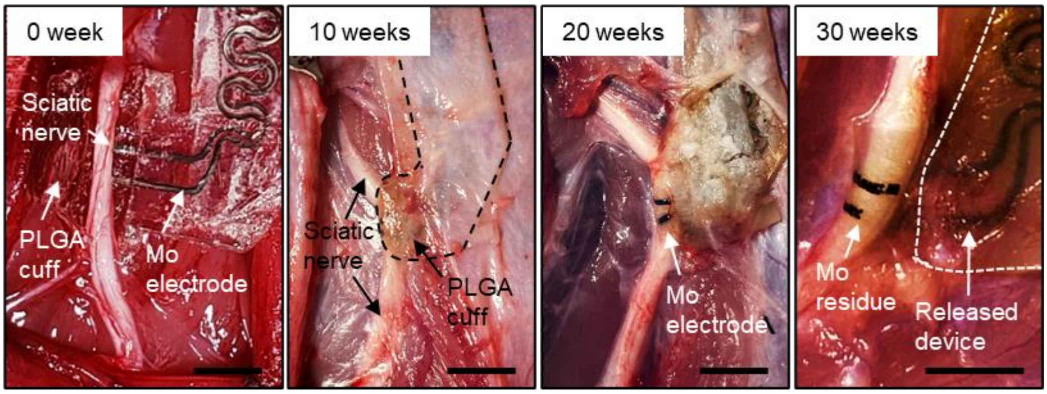

Supplementary Fig. 18 | Photographs of cuff degradation.

Images of the cuff electrode, consisting of PLGA 75:25 (lactide:glycolide) and Mo, on the sciatic nerve for 30 weeks. (*n* = 3 independent animals per group.) The photograph at 0 weeks shows the slightly oversized PLGA cuff electrodes wrapped on the sciatic nerve at the implantation. The photograph at 10 weeks shows that the PLGA cuff and Mo electrode remain wrapped the sciatic nerve securely. The photograph at 20 weeks reveals the residue of the PLGA cuff undergoing bioresorption, and the size of PLGA decreasing, exposing the Mo electrode. At 30 weeks, the disappearance of the PLGA cuff decouples the nerve from the b-DCPU encapsulated part of the device. Some Mo residues remain on the surface of the nerve and eventually undergo degradation. Scale bar indicates 5 mm.

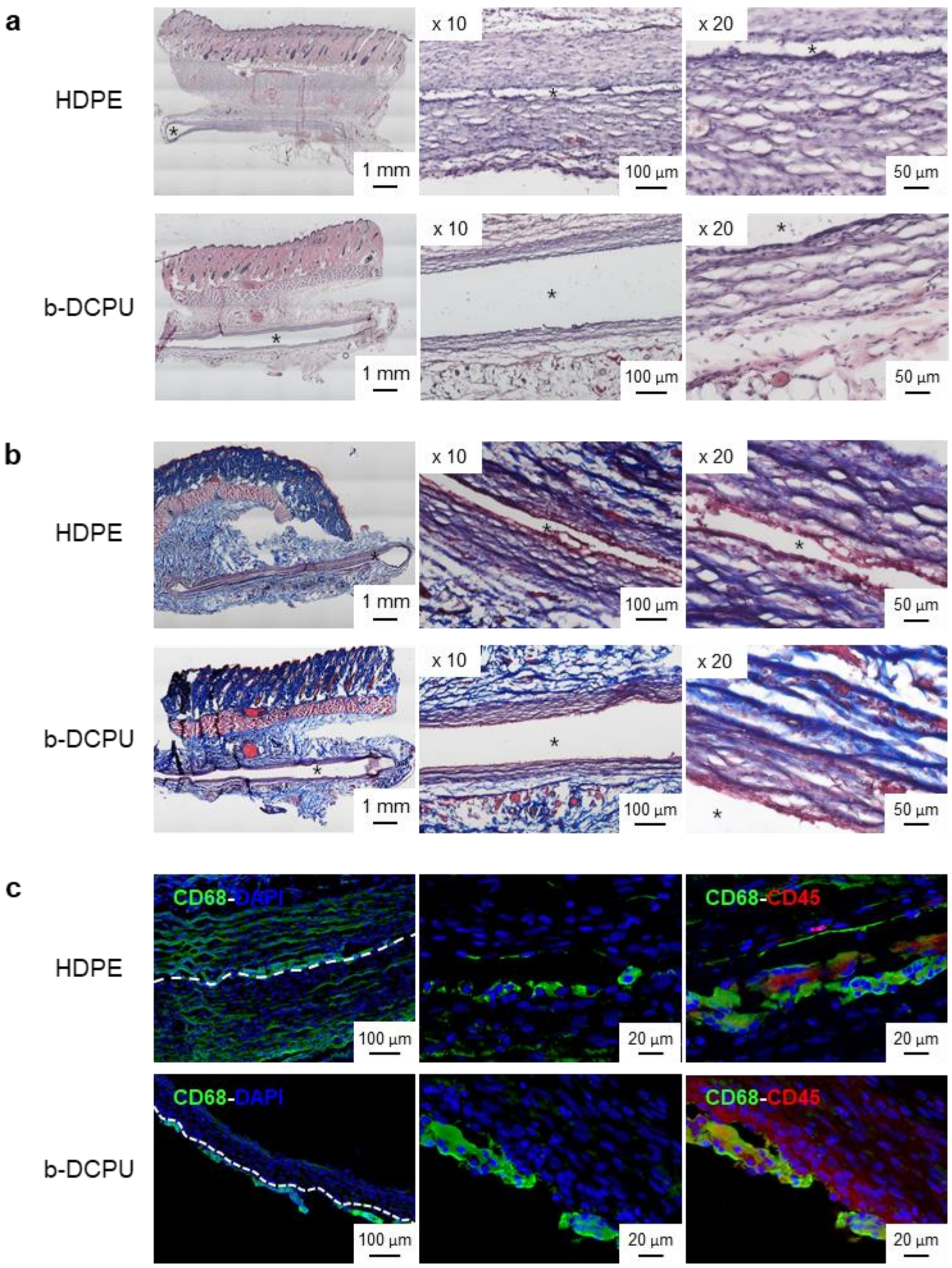

Supplementary Fig. 19 | *In vivo* evaluations of biocompatibility of b-DCPU encapsulation.

We used high density polyethylene (HDPE) as FDA-approved non-toxic control group. **a,** Histological results of H&E staining. **b,** Masson Trichrome staining. **c,** Immunohistochemical staining of skin after two weeks following implantation demonstrate identical monocyte (cd68) and leukocyte (cd45) inflammatory cell infiltration at the material-tissue interface. In **a, b,** and **c**, n = 3 repeated independently with similar results.

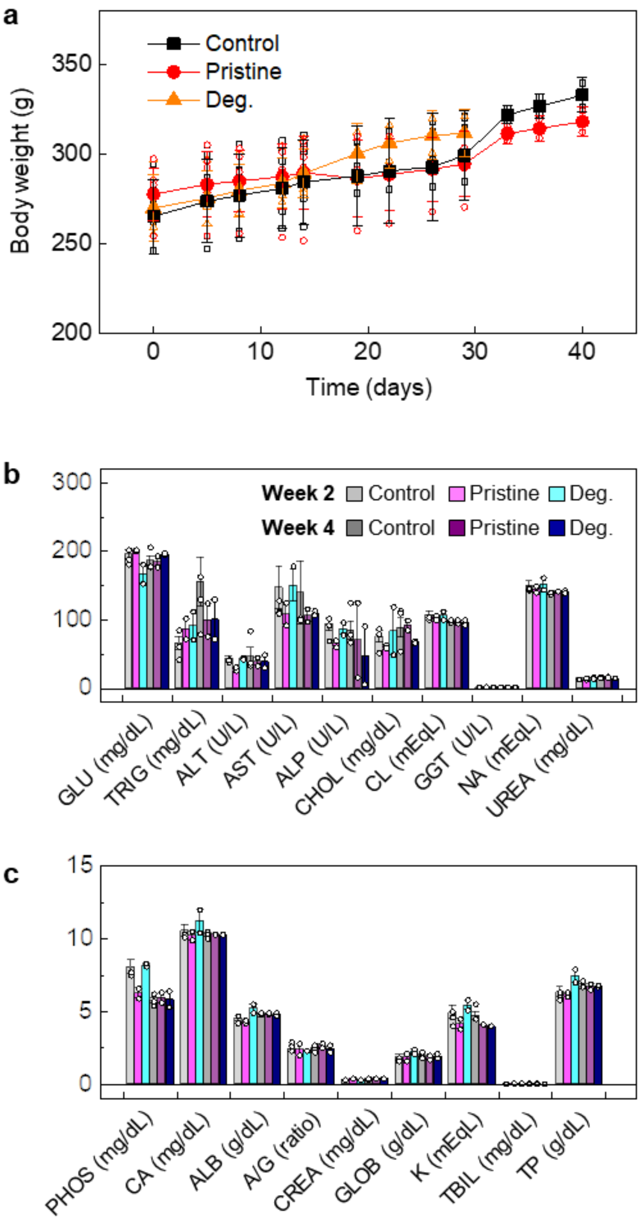

Supplementary Fig. 20 | In vivo biocompatibility evaluations of b-DCPU.

a, Bodyweight in each group measured weekly. *n* = 6 biologically independent animals per groups. Control indicates mice with PLGA implants. Pristine indicates mice with b-DCPU implants without accelerated degradation process in PBS. Degraded group (Deg.; orange) indicates mice with gel-textured b-DCPU implants with accelerated degradation in PBS (95°C) for 1 month. **b, c,** Analysis of complete blood counts and blood chemistry for the rats with and without implantation of b-DCPU. *n* = 2 biologically independent rat. GLU, glucose (mg dL^{-1}); TRIG, triglycerides (mg dL^{-1}); ALT, alanine aminotransferase (U L^{-1}); AST, aspartate transaminase (U L^{-1}), ALP, alkaline phosphatase (U L^{-1}); CHOL, cholesterol (mg dL^{-1}); Cl, chloride (mEqL); GGT, gamma-glutamyl transferase (U L^{-1}); Na, sodium (mEqL); UREA, urea (mg dL^{-1}); PHOS, phosphorus (mg dL^{-1}); Ca, calcium (mg dL^{-1}); ALB, albumin (g dL^{-1}); A/G, albumin/globulin ratio; CREA, creatinine (mg dL^{-1}); GLOB, globulin (g dL^{-1}); K,

potassium (mEqL); TBIL, total bilirubin (mg dL^{-1}); TP, total protein (g dL^{-1}). In **a, b,** and **c**, the results are shown as means ± s.e.m. Data available in source data file.

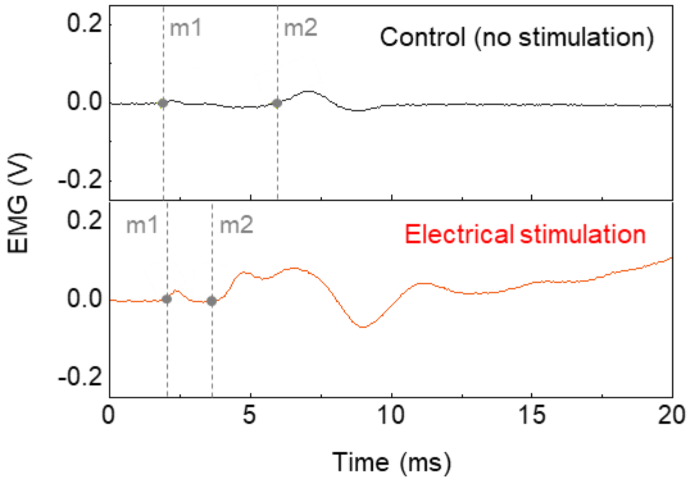

Supplementary Fig. 21 | Electrophysiologic analysis of re-innervated gastrocnemius muscle at 6 weeks.

EMG signals for the control (top, black; no stimulation) and wireless electrical stimulation (bottom, red) groups. Significant increases in the amplitude of compound muscle action potential are observed for the wireless electrical stimulation group, compared to the control group. The distances between m1 and m2 in control and stimulation groups are 4 and 1.75 ms, respectively.

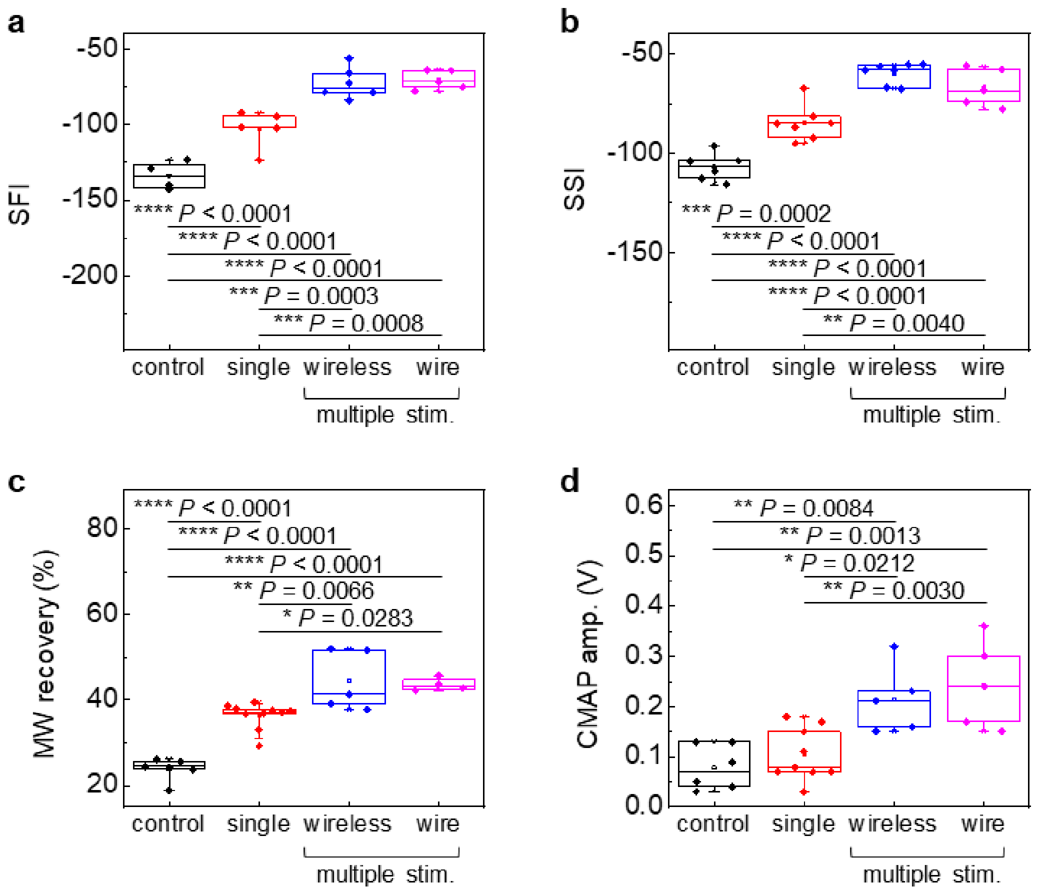

Supplementary Fig. 22 | Comparison studies of the effect of wired and wireless chronic electrical stimulation on functional recovery.

a, Sciatic function index (SFI). *n* = 5 independent animals per group. **b,** Static sciatic index (SSI). *n* = 6 independent animals per group. **c,** Relative muscle weight (MW) recovery. *n* = 5 independent animals per group. **d,** Compound muscle action potential (CMAP). *n* = 5 independent animals per group. The boxplots show the median (center line), the third and first quartiles (upper and lower edge of the box, respectively), and the largest and smallest value that is ≤ 1.5 times the interquartile range (the limits of the upper and lower whiskers, respectively). Statistical software (Version 6.0) was used for the statistical analysis followed by a t-test and one-way ANOVA with Tukey multiple comparison analysis (*: p<0.05; **: p<0.01; ***: p<0.001; ****: p<0.0001). Data available in source data file.

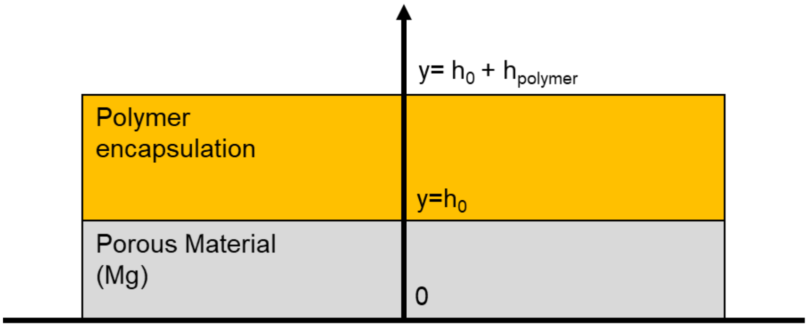

Supplementary Fig. 23 | Schematic illustration of double-layer reactive diffusion with organic encapsulation.

In this model, the single Mg layer thickness (h_0) and the polymer/Mg specimen thickness ($h_0 + h_{\mathrm{polymer}}$), where h_0 and h_{polymer} are the initial thicknesses of the Mg layer and polymer, respectively.

Reference

1. Caverly, R. H. & Hiller, G. The frequency-dependent impedance of p-i-n diodes. *IEEE Trans. Microw. Theory Tech.* **37**, 787–790 (1989).

2. Shin, J. *et al.* Bioresorbable pressure sensors protected with thermally grown silicon dioxide for the monitoring of chronic diseases and healing processes. *Nat. Biomed. Eng.* **3**, 37–46 (2019).

3. Koo, J. *et al.* Wireless bioresorbable electronic system enables sustained nonpharmacological neuroregenerative therapy. *Nat. Med.* **24**, 1830–1836 (2018).

4. Won, S. M. *et al.* Natural Wax for Transient Electronics. *Adv. Funct. Mater.* **28**, 1801819 (2018).

5. Hwang, S. W. *et al.* High-performance biodegradable/transient electronics on biodegradable polymers. *Adv. Mater.* **26**, 3905–3911 (2014).

6. Kang, S. K. *et al.* Dissolution behaviors and applications of silicon oxides and nitrides in transient electronics. *Adv. Funct. Mater.* **24**, 4427–4434 (2014).

7. Kang, S. K. *et al.* Biodegradable thin metal foils and spin-on glass materials for transient electronics. *Adv. Funct. Mater.* **25**, 1789–1797 (2015).

8. Choi, Y. S., Koo, J. & Rogers, J. A. Inorganic materials for transient electronics in biomedical applications. *MRS Bull.* **45**, 103–112 (2020).

9. Tao, H. *et al.* Silk-based resorbable electronic devices for remotely controlled therapy and in vivo infection abatement. *Proc. Natl. Acad. Sci. U. S. A.* **111**, 17385–17389 (2014).

10. Hwang, S.-W. *et al.* A Physically Transient Form of Silicon Electronics. *Science* **337**, 1640–1644 (2012).

11. Luo, M., Martinez, A. W., Song, C., Herrault, F. & Allen, M. G. A microfabricated wireless RF pressure sensor made completely of biodegradable materials. *J. Microelectromechanical Syst.* **23**, 4–13 (2014).

12. Feig, V. R., Tran, H. & Bao, Z. Biodegradable Polymeric Materials in Degradable Electronic Devices. *ACS Cent. Sci.* **4**, 337–348 (2018).

13. Hwang, S. W. *et al.* Biodegradable elastomers and silicon nanomembranes/nanoribbons for stretchable, transient electronics, and biosensors. *Nano Lett.* **15**, 2801–2808 (2015).

14. Scheutz, G. M., Lessard, J. J., Sims, M. B. & Sumerlin, B. S. Adaptable Crosslinks in Polymeric Materials: Resolving the Intersection of Thermoplastics and Thermosets. *J. Am. Chem. Soc.* **141**, 16181–16196 (2019).

15. Zou, W., Dong, J., Luo, Y., Zhao, Q. & Xie, T. Dynamic Covalent Polymer Networks: from Old Chemistry to Modern Day Innovations. *Adv. Mater.* **29**, 1606100 (2017).

16. Menon, A. V., Madras, G. & Bose, S. The journey of self-healing and shape memory polyurethanes from bench to translational research. *Polym. Chem.* **10**, 4370–4388 (2019).

17. Rowan, S. J., Cantrill, S. J., Cousins, G. R. L., Sanders, J. K. M. & Stoddart, J. F.

Dynamic Covalent Chemistry. *Angew. Chemie Int. Ed.* **41**, 1460 (2002).

18. Zhang, Z. P., Rong, M. Z. & Zhang, M. Q. Polymer engineering based on reversible covalent chemistry: A promising innovative pathway towards new materials and new functionalities. *Prog. Polym. Sci.* **80**, 39–93 (2018).

19. Yin, L. *et al.* Dissolvable metals for transient electronics. *Adv. Funct. Mater.* **24**, 645–658 (2014).

20. Yoon, J.-S., Jung, H.-W., Kim, M.-N. & Park, E.-S. Diffusion coefficient and equilibrium solubility of water molecules in biodegradable polymers. *J. Appl. Polym. Sci.* **77**, 1716–1722 (2000).

21. Fleming, P. *et al.* Strain on the human sciatic nerve in vivo during movement of the hip and knee. *J. Bone Jt. Surg. - Ser. B* **85**, 363–365 (2003).

22. Boyd, B. S., Puttlitz, C., Gan, J. & Topp, K. S. Strain and excursion in the rat sciatic nerve during a modified straight leg raise are altered after traumatic nerve injury. *J. Orthop. Res.* **23**, 764–770 (2005).

23. da Silva, G. R., da Silva-Cunha, A., Behar-Cohen, F., Ayres, E. & Oréfice, R. L. Biodegradation of polyurethanes and nanocomposites to non-cytotoxic degradation products. *Polym. Degrad. Stab.* **95**, 491–499 (2010).

24. Wang, J. *et al.* Synthesis and characterization of biodegradable polyurethanes based on L-cystine/cysteine and poly(ϵ-caprolactone). *J. Appl. Polym. Sci.* **128**, 4047–4057 (2013).

25. Mondal, S. & Martin, D. Hydrolytic degradation of segmented polyurethane copolymers for biomedical applications. *Polym. Degrad. Stab.* **97**, 1553–1561 (2012).

26. Kupka, V., Vojtova, L., Fohlerova, Z. & Jancar, J. Solvent free synthesis and structural evaluation of polyurethane films based on poly(Ethylene glycol) and poly(caprolactone). *Express Polym. Lett.* **10**, 479–492 (2016).

27. Chiono, V. *et al.* Synthetic biodegradable medical polyurethanes. in *Science and Principles of Biodegradable and Bioresorbable Medical Polymers: Materials and Properties* 189–216 (2017). doi:10.1016/B978-0-08-100372-5.00006-4.

28. Doucet, B. M., Lam, A. & Griffin, L. Neuromuscular electrical stimulation for skeletal muscle function. *Yale J. Biol. Med.* **85**, 201–215 (2012).

29. Maffiuletti, N. A. *et al.* Clinical Use of Neuromuscular Electrical Stimulation for Neuromuscular Rehabilitation: What Are We Overlooking? *Arch. Phys. Med. Rehabil.* **99**, 806–812 (2018).

30. Bergquist, A. J., Wiest, M. J. & Collins, D. F. Motor unit recruitment when neuromuscular electrical stimulation is applied over a nerve trunk compared with a muscle belly: quadriceps femoris. *J. Appl. Physiol.* **113**, 78–89 (2012).

31. Lozano, R. *et al.* Electrical stimulation enhances the acetylcholine receptors available for neuromuscular junction formation. *Acta Biomater.* **45**, 328–339 (2016).

32. Willand, M. P. *et al.* Daily Electrical Muscle Stimulation Enhances Functional Recovery Following Nerve Transection and Repair in Rats. *Neurorehabil. Neural Repair* **29**, 690–700 (2014).

33. Cheetham, J. *et al.* Effects of Functional Electrical Stimulation on Denervated Laryngeal Muscle in a Large Animal Model. *Artif. Organs* **39**, 876–885 (2015).

34. Sheffler, L. R. & Chae, J. Neuromuscular electrical stimulation in neurorehabilitation. *Muscle Nerve* **35**, 562–590 (2007).

35. Boutry, C. M. *et al.* Towards biodegradable wireless implants. *Philos. Trans. R. Soc. A Math. Phys. Eng. Sci.* **370**, 2418–2432 (2012).

36. Maytin, M. & Epstein, L. M. Lead extraction is preferred for lead revisions and system upgrades when less is more. *Circ. Arrhythmia Electrophysiol.* **3**, 413–424 (2010).

37. Li, R. *et al.* An analytical model of reactive diffusion for transient electronics. *Adv. Funct. Mater.* **23**, 3106–3114 (2013).

38. Moldovan, M., Alvarez, S. & Krarup, C. Motor axon excitability during Wallerian degeneration. *Brain* **132**, 511–523 (2009).

39. Conforti, L. *et al.* NAD+ and axon degeneration revisited: Nmnat1 cannot substitute for WldS to delay Wallerian degeneration. *Cell Death Differ.* **14**, 116–127 (2007).

40. Babetto, E. *et al.* Targeting NMNAT1 to Axons and Synapses Transforms Its Neuroprotective Potency In Vivo. *J. Neurosci.* **30**, 13291–13304 (2010).

41. Gilliatt, R. W. & Hjorth, R. J. Nerve conduction during Wallerian degeneration in the baboon. *J. Neurol. Neurosurg. & Psychiatry* **35**, 335–341 (1972).

42. Rotshenker, S. Wallerian degeneration: the innate-immune response to traumatic nerve injury. *J. Neuroinflammation* **8**, 109 (2011).

43. Chaudhry, V. & Cornblath, D. R. Wallerian degeneration in human nerves: Serial electrophysiological studies. *Muscle Nerve* **15**, 687–693 (1992).

44. Kamble, N., Shukla, D. & Bhat, D. Peripheral Nerve Injuries: Electrophysiology for the Neurosurgeon. *Neurol. India* **67**, 1419–1422 (2019).
